# MEBOCOST maps metabolite-mediated intercellular communications using single-cell RNA-seq

**DOI:** 10.1093/nar/gkaf569

**Published:** 2025-06-26

**Authors:** Rongbin Zheng, Yang Zhang, Tadataka Tsuji, Xinlei Gao, Farnaz Shamsi, Allon Wagner, Nir Yosef, Kui Cui, Hong Chen, Michael A Kiebish, Juan J Aristizabal-Henao, Niven R Narain, Lili Zhang, Yu-Hua Tseng, Kaifu Chen

**Affiliations:** Department of Cardiology, Boston Children's Hospital, Boston, MA, 02115, United States; Department of Pediatrics, Harvard Medical School, Boston, MA, 02115, United States; Section on Integrative Physiology and Metabolism, Joslin Diabetes Center, Harvard Medical School, Boston, MA, 02115, United States; Department of Medicine, Harvard Medical School, Boston, MA, 02115, United States; Section on Integrative Physiology and Metabolism, Joslin Diabetes Center, Harvard Medical School, Boston, MA, 02115, United States; Department of Medicine, Harvard Medical School, Boston, MA, 02115, United States; Department of Cardiology, Boston Children's Hospital, Boston, MA, 02115, United States; Department of Pediatrics, Harvard Medical School, Boston, MA, 02115, United States; Section on Integrative Physiology and Metabolism, Joslin Diabetes Center, Harvard Medical School, Boston, MA, 02115, United States; Department of Medicine, Harvard Medical School, Boston, MA, 02115, United States; Department of Electrical Engineering and Computer Sciences, University of California, Berkeley, Berkeley CA, 94720, United States; Department of Molecular and Cell Biology, University of California, Berkeley, Berkeley CA, 94720, United States; Center for Computational Biology, University of California, Berkeley, Berkeley CA, 94720, United States; Department of Electrical Engineering and Computer Sciences, University of California, Berkeley, Berkeley CA, 94720, United States; Department of Molecular and Cell Biology, University of California, Berkeley, Berkeley CA, 94720, United States; Center for Computational Biology, University of California, Berkeley, Berkeley CA, 94720, United States; Department of Systems Immunology, Weizmann Institute of Science, Rehovot, 76100, Israel; Vascular Biology Program, Boston Children's Hospital and Department of Surgery, Harvard Medical School, Boston, MA, 02115, United States; Department of Surgery, Harvard Medical School, Boston, MA, 02115, United States; Vascular Biology Program, Boston Children's Hospital and Department of Surgery, Harvard Medical School, Boston, MA, 02115, United States; Department of Surgery, Harvard Medical School, Boston, MA, 02115, United States; BPGbio, Framingham, MA, 01701, United States; BPGbio, Framingham, MA, 01701, United States; BPGbio, Framingham, MA, 01701, United States; Department of Cardiology, Boston Children's Hospital, Boston, MA, 02115, United States; Department of Pediatrics, Harvard Medical School, Boston, MA, 02115, United States; Section on Integrative Physiology and Metabolism, Joslin Diabetes Center, Harvard Medical School, Boston, MA, 02115, United States; Department of Medicine, Harvard Medical School, Boston, MA, 02115, United States; Department of Cardiology, Boston Children's Hospital, Boston, MA, 02115, United States; Department of Pediatrics, Harvard Medical School, Boston, MA, 02115, United States

## Abstract

Cell-cell communication (CCC) is crucial for cellular function and tissue homeostasis. Existing methods for protein-oriented CCC detection often overlook metabolite-mediated CCC (mCCC), and adapting them to mCCC analysis is challenging due to fundamental differences in the underlying biological mechanisms. To fill this gap, we developed MEBOCOST, an algorithm built on scRNA-seq and metabolic flux balance analysis to detect mCCC among single cells. Comprehensive benchmarking analyses based on simulation, spatial, CRISPR screen, and clinical patient data demonstrated the robustness of MEBOCOST in detecting biologically significant mCCC events. We applied MEBOCOST to scRNA-seq datasets of human white adipose tissues and unraveled macrophages were the predominant source of mCCC reprogramming in obese patients. Moreover, analysis in mice brown adipose tissue successfully recapitulated known and further uncovered new mCCC events, including a glutamine-mediated endothelial-to-adipocyte communication, which was experimentally verified to regulate adipocyte differentiation. Therefore, MEBOCOST is a valuable tool for researchers investigating mCCC in diverse biological contexts and disease samples. MEBOCOST is freely available at https://github.com/kaifuchenlab/MEBOCOST.

## Graphical Abstract

**Graphical Abstract:**
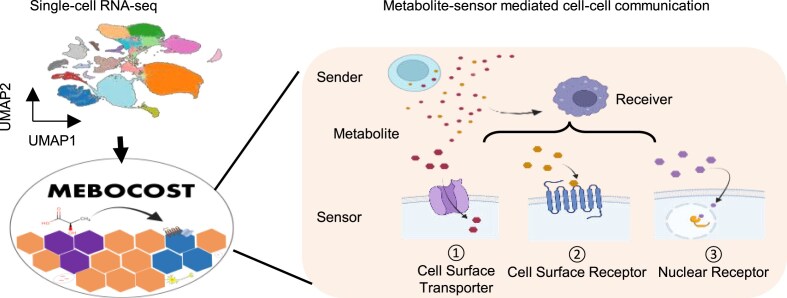

## Introduction

Cell-cell communication (CCC) is crucial for maintaining the functions and homeostasis of cells, organs, and intact systems [1]. Abnormal CCC has been shown to contribute to many health anomalies, such as obesity [2], diabetes [3], heart disease [4], and cancer [5]. CCC can be mediated by various types of molecules, e.g. proteins and metabolites. Single-cell RNA sequencing (scRNA-seq) data provides gene expression profiles of single cells in a tissue, including those of signaling molecules, which offers the opportunity to model CCC between cells in a tissue microenvironment. Previous studies modeling CCC based on scRNA-seq have been extensively focused on protein-mediated ligand–receptor interactions. Some great examples include applications of CellPhoneDB, CellChat, and NicheNet [6–8]. However, inferring metabolite-mediated CCC (mCCC) has been challenging. In an era where we can access millions of cells in scRNA-seq data, this gap limits the systematic investigation of intercellular crosstalk in healthy and diseased tissues.

Growing evidence has been accumulated on the critical roles of metabolites as signaling molecules that regulate various biological functions. During the mCCC process, metabolites produced by sender cells travel to receiver cells, where those metabolites bind to sensor proteins in receiver cells and trigger the regulation of biological processes [9, 10]. It is widely reported that mCCC can regulate various cellular functions. For instance, polyamines produced and secreted by endothelial cells have been reported to be sensed by β-adrenergic receptors on the surface of white adipocytes, thereby regulating adiposity [11]. Acetylcholine derived from B lymphocytes has been reported to limit hematopoiesis by interacting with cholinergic nicotinic receptor α7 (Chrna7) on endothelial and mesenchymal stromal cells in bone marrow [12]. Further, mCCC plays a critical role in diseases such as cancer. Li *et al.* reported that interactions between tumoral histamine and histamine receptor H1 (HRH1) of macrophages rendered T cell dysfunction [13]. Zhang *et al.* revealed that the B cell-derived neurotransmitter GABA promotes monocyte differentiation into anti-inflammatory macrophages, thus limiting anti-tumor immunity by inhibiting CD8 T cell killing [14]. However, the lack of reliable tools for systematically identifying mCCC has been significantly constrained in mapping and investigating metabolites mediating intercellular communications in different biological scenarios.

The method to analyze mCCC using scRNA-seq data would root a fundamental difference in the underlying biological mechanism compared to that of CCC mediated by protein ligands and receptors. First, the receiver cells in mCCC can have different sensor proteins to mediate the signaling metabolites, including cell surface receptors [15], cell surface transporters [16], and nuclear receptors [17]. For those mCCC mediated by cell surface transporters and nuclear receptors, accounting for the uptake of metabolites is essential for identifying receiver cells. This contrasts with protein ligand–receptor interactions, where such consideration is unnecessary since they exclusively involve cell surface receptors. Second, mCCC analysis needs to integrate metabolic networks due to the complexity of metabolism [18]. A few studies have adapted methods from protein-mediated CCC analysis to analyze mCCC [19–21]. However, such an adaption is challenging because methods for protein-mediated CCC analysis typically ignore secretion and metabolic networks [6–8, 22, 23]. Analysis of mCCC also differs from cell-cell metabolic reaction analysis, which infers that enzymes in a receiver cell consume a metabolite produced by other enzymes in a sender cell [24]. Conversely, most metabolites received by receiver cells are not typically converted into another type by the sensor protein in mCCC, particularly for those interactions mediated by cell surface and nuclear receptors. Consequently, existing algorithms designed to compute fluxes of metabolites during cell-cell metabolic reactions overlook those events mediated by cell surface and nuclear receptors in mCCC [25]. Current challenges in the systematic analysis of mCCC include the need for a robust computational method to detect active mCCC events in a sample.

The flux balance analysis (FBA) is a well-established statistical method for calculating the flow of metabolites through the metabolic networks in the genome-scale metabolic models (GEM) [26]. Integrating the FBA with gene expression data allows for investigating sample-specific metabolic flux [27, 28], including metabolite secretion and uptake, which are crucial for an mCCC process. In this study, we first present MEBOCOST, a bioinformatics technology developed on top of scRNA-seq and metabolic flux analysis to detect mCCC. We benchmarked the performance of MEBOCOST using simulation, spatial, CRISPR screen, and patient datasets, which showed the robustness of MEBOCOST in detecting biologically meaningful mCCC events. Moreover, MEBOCOST unraveled mCCC changes within human white adipose tissue (WAT) during the development of obesity. We pinpointed distinct mCCC events in WAT of high body mass individuals compared to those with low body mass, showing that macrophages and vascular cells were the predominant cell types involved in these specific events. By applying MEBOCOST to mouse brown adipose tissue (BAT) during thermogenesis, we successfully identified known mCCC and revealed new mCCC experimentally verified to regulate adipocyte differentiation. Therefore, MEBOCOST provides a valuable resource for gaining new insights into metabolic signaling as a molecular basis of diverse developmental and disease processes.

## Materials and methods

### Collection of extracellular metabolites, enzyme genes, and metabolite-sensor partners

MEBOCOST was developed to integrate prior knowledge of metabolite-sensor partners with scRNA-seq data in the algorithm. To this end, we collected extracellular metabolites, related enzymes, and metabolite-sensor partners from public databases and literature. All metabolites were mapped to HMDB standard names to obtain uniform annotations. The metabolites in “extracellular space,” “blood,” or “cerebrospinal fluid” were defined as “extracellular metabolites” and were used by MEBOCOST. The metabolite-enzyme associations were collected for extracellular metabolites from the HMDB database. Three types of sensor proteins were focused in this study, namely, cell surface transporter, cell surface receptor, and nuclear receptors. A workflow combining computational text-mining and manual collection was developed to collect known pairs of metabolite-sensor partners from PubMed abstracts. The detailed procedures can be found in Supplementary Methods. Briefly, we check all potential combinations of metabolite and sensor names in each context of publication titles, MeSH words, and abstracts. The metabolite-sensor pairs were collected if metabolite and sensor names were co-mentioned in the same sentence and were subjected to manual curation later. We manually collected metabolite-sensor partners from well-known databases such as the HMDB [29–32], Recon2 [33], GPCRdb [15, 34], Wikipedia, and GeneCards [35]. All the metabolite-sensor partners were manually curated by at least three curators separately.

### Development of the MEBOCOST algorithm

With scRNA-seq as input, MEBOCOST first aggregated gene expression of metabolite enzymes based on the collected metabolite and enzyme relationships. Many metabolites participate in multiple reactions. Some reactions take the metabolites of interest as substrates (consuming reactions), while others may take those metabolites as products (producing reactions). The two types of metabolic reactions dynamically influence the level of a metabolite. Therefore, the gene expression of metabolite enzymes was aggregated by calculating the average expression of enzymes in producing reactions and subtracting the average expression of enzymes in consuming reactions. Note that this is a qualitative filtering of metabolites based on the enzyme expression rather than a quantitative estimation of metabolite abundance. Next, MEBOCOST identified significant co-expression of metabolite enzyme and sensor among the cell populations in the scRNA-seq data. Briefly, MEBOCOST computed an enzyme-sensor co-expression score for each metabolite-sensor partner between each pair of cell groups. A permutation test was performed to determine the significance of an enzyme-sensor co-expression score [36], followed by the false discovery rate (FDR) [37]. MEBOCOST computed the communication score as the co-expression score of metabolite enzyme and sensor pairs, normalized by the mean background co-expression score obtained from the random shuffling procedure.

Lastly, MEBOCOST allows users to integrate metabolic flux analysis to constrain the metabolite secretion further and uptake potential in mCCC analysis. While it is challenging to model the flux between two cell types, several tools have been published to model the metabolic flux for each cell type or single cell. For example, scFEA [38] was reported to estimate cell-wise metabolic flux for both balance and imbalance metabolism systems. The COMPASS [27] was developed to characterize cellular metabolic states based on scRNA-seq and FBA. The scFBA [25] was published for cancer metabolism analysis by integrating scRNA-seq into population models. The inferred metabolic fluxes by those tools include metabolite effluxes from cells to the extracellular spaces and metabolite influx from extracellular space to receiver cells. Users can integrate flux results from these and any other estimators of interest if the metabolite efflux and influx matrix can be provided to the MEBOCOST. The integration with FBA results improved the mCCC analysis by further defining a sender if the metabolite efflux exceeds a threshold. Similarly, a receiver was determined if the influx score surpassed a threshold, with the default setting for the 25^th^ percentile of all efflux or influx scores. The detailed design of the MEBOCOST algorithm can be found in the Supplementary Methods.

### Single-cell RNA sequencing and data analysis

The single-cell RNA-seq data of stromal vascular fraction (SVF) of mouse brown adipose tissue from TN (30°C for 7 days), RT (22°C), Cold2 (5°C for 2 days), and Cold7 (5°C for 7 days) was generated by 10X Genomics platform [39, 40] using 9-week-old male C57BL/6J mice.

For data analysis, cellranger was applied to map the raw sequence to the mouse reference genome (mm10) and obtain the read count over each gene. Scanpy was used for downstream processing, including normalization, dimension reduction, clustering, and visualization [41]. The cell type marker genes of PanglaoDB [42] were used for cell type annotation. The detailed data analysis can be found in the Supplementary Methods.

### Integration of public datasets

Several public datasets were used in this study. The public scRNA-seq datasets were used, including the data in white adipose [43], colon tumor, heart [44], intestine [45], pancreatic tumor [46], and squamous cell carcinoma [47]. The processed gene expression matrix and cell annotation table were obtained from the Single Cell Portal (SCP1376), TISCH database (“CRC_GSE146771_Smartseq2”), and the publication of Liu *et al.* [48]. We also obtained spatial transcriptomics (ST) data on the Visium platform from Liu *et al.* [48]. To integrate with the scRNA-seq data in the same tissue type, STRIDE [49] was used to deconvolute the proportion of cell types in every spot of the ST sample. The deconvoluted data were used to compute the colocalization score for any two cell types as the Spearman correlation of their proportions across all spots, following methodologies described in prior studies [50]. A CRISPR screen data with the HCT15 and NK coculture (BioGRID ORCS, ID 1657) was analyzed using the gene level statistics computed by the MAGeCK RRA algorithm [51]. TCGA colorectal tumor samples from UCSC XENA (“TCGA Colon and Rectal Cancer (COADREAD) (15 datasets)”) were downloaded for the patient survival analysis based on the product of expression values of the aggerated metabolite enzyme and sensor genes. The detailed data analysis can be found in the Supplementary Methods.

### Experimental validation of L-glutamine mediated mCCC

To validate the secretion of L-glutamine by endothelial cells, mouse endothelial cells (ATCC, Cat# C166) were cultured in glutamine-free DMEM for 24 h. The supernatant was collected for glutamine measurement using a Glutamine Assay Kit (CELL BIOLABS, INC. Cat# MET-5165). The relative fluorescence units were read and converted to glutamine concentrations in μM following the instructions provided by the kit. Glutamine-free DMEM supplemented with 10% dialyzed FBS was used as background for the measurement.

The cell proliferation and differentiation analysis were performed to validate the effect of L-glutamine on brown adipocytes. The primary adipocyte progenitor cells from SVF of interscapular BAT in C57BL6 mice were collected as previously described [40]. They were cultured with glutamine-free DMEM supplemented with vehicle or 300 μM L-glutamine in the cell proliferation analysis. For differentiation analysis, immortalized brown preadipocytes were used. To assess the effect of glutamine on differentiation, the cells were treated with 300 μM L-glutamine and a control vehicle, respectively. The adipocyte differentiation was evaluated by quantifying the lipid accumulation level using Oil Red O staining. Further, qRT-PCR was performed to quantify the mRNA expression of brown adipocyte marker genes, including *Fabp4*, *Pparg*, and *Prdm16*. To validate the L-glutamine transporters in brown adipocytes, the SLC1A5 and SLC38A2 were knocked down via CRISPR-Cas9 technique and inhibited by a small molecular inhibitor VP302 (10 μM, SelleckChem Cat# S8818). The proliferation or differentiation analysis was performed as the same procedure for transporters-perturbed cells.

Overexpression of *Glul* in endothelial cells was performed to validate the positive association between L-glutamine enzyme and secretion. To this end, the *Glul* overexpression plasmid was constructed and packaged into Lentiviral particles. Endothelial cells were infected with lentivirus expressing *Glul* and selected with Geneticin selective antibiotic for 7 days to overexpress *Glul*. An empty vector was used as a control. Western blotting was performed to confirm the overexpression of GLUL protein. The detailed experimental procedures can be found in the Supplementary Methods.

### Quantification and statistical analysis

Statistical analyses were performed with Python 3.8. T-tests, U-tests, or Wilcoxon tests were used as appropriate and indicated in the figure legends. The Spearman rank correlation coefficient was used to measure the correlations. The data are presented as mean in bar plots or individual points with box plots or violin plots that display the median and quartiles. Error bars represent standard error around the mean in bar plots. The sample sizes represented in all plots were labeled in the figure legends.

## Results

### Developing MEBOCOST on top of scRNA-seq and FBA techniques to detect mCCC

**Figure 1.**
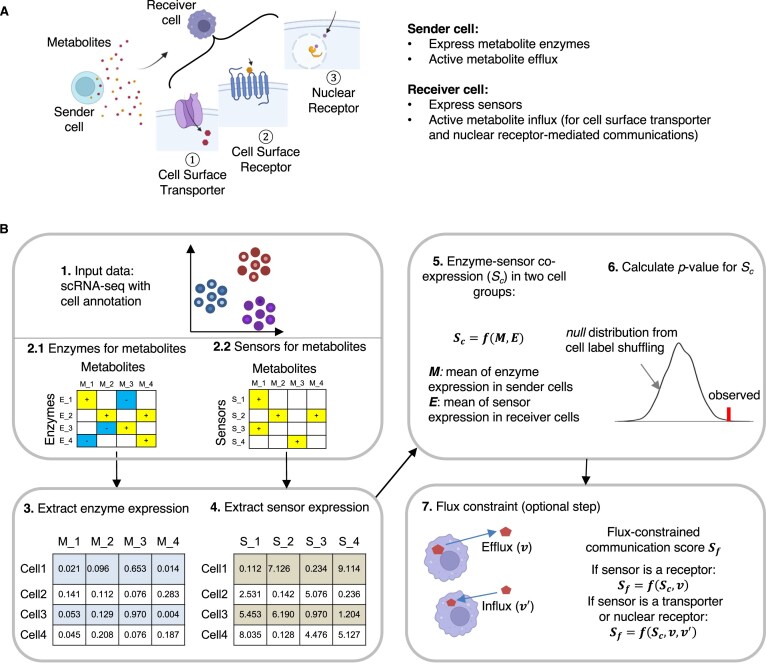
MEBOCOST detects metabolite-sensor communications between cells with scRNA-seq. (**A**) Cartoon to show that MEBOCOST predicts metabolite-mediated cell-cell communications, in which sender cells secret metabolites and receiver cells receive metabolites through three types of sensor proteins, including cell surface transporter, cell surface receptor, and nuclear receptor. (**B**) Cartoons to show the seven steps in MEBOCOST to identify cell-cell metabolite-sensor communications. [1] The RNA expression data and cell type annotation obtained from scRNA-seq were taken as the input data to MEBOCOST. [2] A knowledgebase of enzymes (2.1) and sensor proteins (2.2) for metabolites is incorporated into the MEBOCOST algorithm. [3] The RNA expression values of the metabolite enzymes were extracted from scRNA-seq data. [4] The RNA expression values of sensor proteins for metabolites were extracted from the scRNA-seq data. [5] Calculate the enzyme-sensor co-expression score between two cell groups by taking the product of the mean of enzyme expression values in the sender cells and the mean of sensor expression values in the receiver cells. [6] Shuffling single cell labels to generate a statistical *null* distribution to calculate the *P*-value for the enzyme-sensor co-expression score. [7] Incorporating metabolite efflux rates to indicate secretion in sender cells and influx rates to indicate uptake activity in receiver cells, respectively, for combining with the enzyme-sensor co-expression score to identify communication events. All cartoons in this figure were created using BioRender.

Many metabolites can function as signaling molecules [9, 15, 52] in the extracellular space and interact with sensor proteins of nearby cells to form mCCC. To facilitate single-cell metabolism research, we developed MEBOCOST, a computational method for mCCC identification between a pair of sender and receiver cell groups. The sender cell population exhibits gene expression of biosynthetic enzymes and active metabolite efflux. The receiver cells were those with sensor expression and, further, with active metabolite influx when the sensors are cell surface transporters or nuclear receptors rather than cell surface receptors (Fig. 1A).

MEBOCOST integrates metabolic flux analysis and single-cell RNA expression information with our manually curated database of extracellular metabolites, metabolic enzymes, and metabolite-sensor partners to detect mCCC (Fig. 1B steps 1–2). To this end, MEBOCOST first extracts RNA expression values of the enzymes and sensors from the scRNA-seq data (Fig. 1B steps 3–4, Supplementary Fig. S1). The genes encoding enzymes of extracellular metabolites were collected from the Human Metabolome Database (HMDB) [29–31, 32]. The extracted values were averaged per cell group to compute a metabolite enzyme and sensor co-expression score. This score is calculated by multiplying the averaged metabolite enzyme expression in the sender cell group with the averaged sensor expression in the receiver cell group (Fig. 1B step 5). The co-expression score served as a constraint to filter out metabolites for which the enzymes or sensors are not highly expressed. MEBOCOST assessed the statistical significance (*P* values) of the co-expression scores based on a permutation test (one-tailed, Fig. 1B step 6) by shuffling the cell labels of all cells in the scRNA-seq data. The *P* values were subjected to the FDR calculation based on Benjamini-Hochberg's method [37]. Several recent single-cell FBA algorithms enabled the evaluation of metabolite effluxes and influxes between extracellular space and a cell based on GEM and scRNA-seq data [25, 27, 38]. MEBOCOST provides the option to integrate FBA results to evaluate metabolite effluxes for secretion in sender cells and influxes for uptake in receiver cells between each pair of cell groups with significant co-expression of enzymes and sensors (Fig. 1B step 7). This evaluation filters out sender and receiver cells with low secretion and uptake fluxes.

### A curated database of enzyme-metabolite-sensor partners for mCCC study

**Figure 2.**
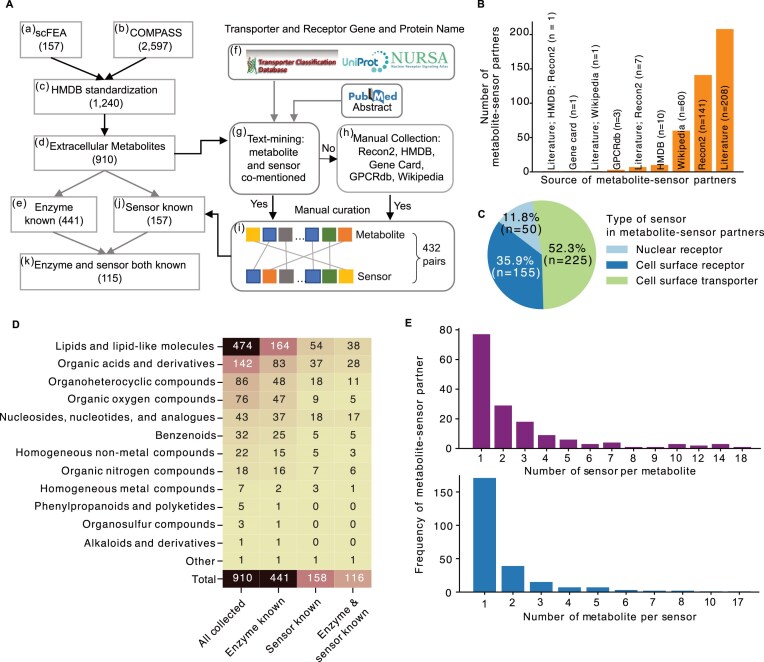
The collection of extracellular metabolites along with their enzymes and sensor proteins. (**A**) The pipeline to collect extracellular metabolites along with their enzymes and sensors. (**B**) Bar plot to show the number of metabolite-sensor partners collected from different sources. (**C**) A pie chart to show the number of each type of sensor protein in the collected metabolite-sensor partners. (**D**) A heatmap to show the number of each type of metabolite in the collected metabolite-sensor partners. (**E**) Number of metabolite-sensor partners plotted against number of sensors per metabolite (top panel) and number of metabolites per sensor (bottom panel).

MEBOCOST starts from metabolites whose flux rates can be inferred based on gene expression data using the metabolic flux analysis software scFEA [38] (157 metabolites) and COMPASS [27] (2,597 metabolites) (Fig. 2A steps a and b). By mapping metabolites to those in the HMDB [30] database, we narrowed the list to 1,240 metabolites, each with a unique accession number (Fig. 2A step c). We then narrowed the list to the 910 metabolites annotated as can be in extracellular space (Fig. 2A step d). We required both enzyme expression and flux rate of metabolites to deliver a reliable inference of mCCC in our model. We thus further narrowed down the total metabolites to 441 with producing enzymes annotated in the HMDB (Fig. 2A step e).

Many studies have reported the critical functions of metabolite-sensor partners. To collect those metabolite-sensor partners thoroughly, we first developed a computational pipeline for comprehensive literature mining. We retrieved 233 transporters from the Transporter Classification Database [53–57], 1,522 cell surface receptor proteins from the UniProt database [58], and 48 nuclear receptors from the Nuclear Receptor Signaling Atlas (NURSA) database [59] (Fig. 2A step f). Next, a text-mining algorithm was developed to parse the metabolite-sensor partners from the abstracts of publications in the PubMed database. An abstract would be selected by the algorithm if any pair of metabolite and sensor were co-mentioned in a sentence of the abstract (Fig. 2A step g). The selected abstracts were then subjected to three rounds of manual curation to confirm the reported metabolite-sensor partners. We read every abstract and filtered out the metabolite-sensor partners for which the evidence in the abstracts was insufficient. We also searched for annotated metabolite-sensor partners in five public databases, including the Recon2 [33], HMDB [29–32], GeneCards [35], GPCRdb [34, 60], and Nuclear Receptor pages of the Wikipedia site (Fig. 2A step h). Together, these procedures resulted in 432 metabolite-sensor partners (Fig. 2A step I, Supplementary Table S1), including 208, 141, 60, and 23 partners from literature, Recon2, Wikipedia, and other multiple sources, respectively (Fig. 2B). Among these partners, 52.3% were metabolite-transporter partners, 35.9% were metabolite-cell surface receptor partners, and 11.8% were metabolite-nuclear receptor partners (Fig. 2C). These metabolite-sensor partners consist of 157 metabolites (Fig. 2A step j). We narrowed the focus to 115 metabolites associated with both enzyme and sensor proteins (Fig. 2A step k). Of these 115 metabolites, a majority (33%) are classified as lipids and lipid-like molecules (Fig. 2D). Organic acids and derivatives also comprise a substantial proportion (25%). Other significant categories include “nucleosides, nucleotides, and analogs” (15%), organoheterocyclic compounds (9%), and 10 other minor types. Most metabolites were associated with one sensor among those collected metabolite-sensor partners. However, some metabolites can interact with multiple sensors (Fig. 2E). Likewise, while most sensors were specific to one metabolite, we identified some sensors that could interact with various metabolites. This metabolite-sensor partner database provides a comprehensive resource for researchers investigating mCCC.

### Orthogonal data showed the biological significance of mCCC detected by MEBOCOST

**Figure 3.**
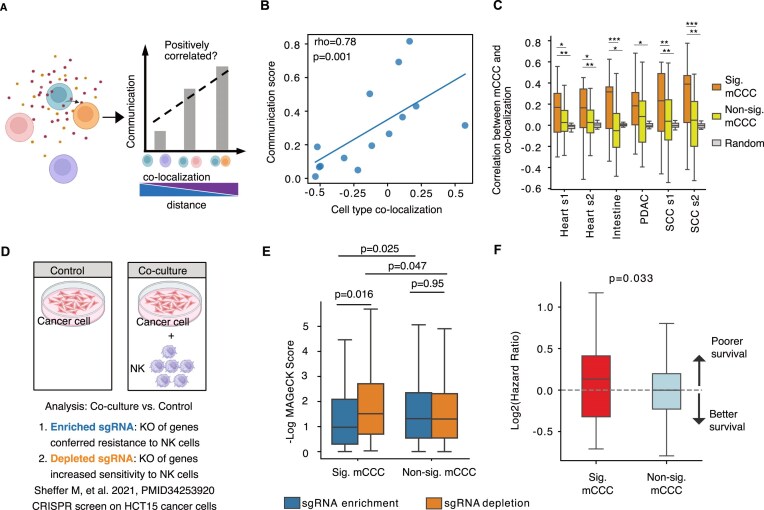
Evaluating the performance of MEBOCOST using orthogonal data. (**A**) A carton to show the rationale of method evaluation using spatial information of the cells. The colocalized or proximal cells were expected to have a higher probability of communication. (**B**) An example to show the correlation between ADP-mediated cell-cell communication score and colocalization score among individual pairs of cell types. Each dot represents a pair of cell types. The solid line showed the regression line. (**C**) Boxplots to show the correlation coefficient between spatial colocalization and communication scores among individual pairs of cell types in each of seven samples. Metabolites that each mediated communications between 10 or more different pairs of cell types were analyzed. SCC, squamous cell carcinoma. PDAC, pancreatic tumor. Suffixes s1 and s2 indicate different samples of the same tissue type. The one-tailed Wilcoxon rank-sum test was used to test the two groups’ statistical differences (*P*-value). This test includes 39, 39, 14, 36, and 21 metabolites for heart s1, heart s2, intestine, PDAC, SCC c1, and SCC s2, respectively. * denotes *P*-value < 0.05, ** denotes *P*-value < 0.01. (**D**) The schematic plot shows the experimental design of a public CRISPR screen dataset from colorectal cancer cells with and without NK cell co-culture. By comparing the sgRNA detected between coculture and control conditions (without co-culture), genes with enriched sgRNA conferred resistance to NK killing upon KO of those genes in cancer cells. Meanwhile, genes with sgRNA depletion suggested increased sensitivity to NK killing upon KO of those genes in cancer cells. (**E**) The boxplot shows the scores for sgRNA depletion (orange box) and enrichment (blue box) for genes (enzymes and sensors) involved in cancer-NK significant and non-significant mCCC. The Y axis shows the -log scores for sgRNA depletion or enrichment computed by the MAGeCK software. Two-tailed Wilcoxon signed-rank test was used to test the statistical difference (*P*-value) between the MAGeCK scores of sgRNA enrichment and depletion for the same gene group (*n* = 98 for genes of cancer-NK sig. mCCC and *n* = 680 for non-sig. mCCC genes). A one-tailed U-test was used to test the difference in MAGeCK scores between the two gene groups. (**F**) A box plot showing the log2 hazard ratio of TCGA patients computed using expression of mCCC-involved genes between those from significant (*n* = 30) and non-significant (*n* = 193) mCCC. A positive log2 hazard ratio suggests that the gene expression is associated with worse patient survival, while a negative log2 hazard ratio suggests that the gene expression is associated with better survival. A one-tailed t-test was used to compute the p-value between the log2 hazard ratio of significant mCCC and non-significant mCCC. All cartoons in this figure were created using BioRender.

It is well recognized that CCC events generally tend to happen between spatially proximal cells [61, 62]. Therefore, researchers have employed ST analysis to provide orthogonal information for evaluating the computational methods that use scRNA-seq data to infer CCC [48] (Fig. 3A). Similarly, we evaluated MEBOCOST by examining the correlation between mCCC and spatial colocalization scores between cell types using datasets from heart [44], intestine [45], pancreatic tumor [46], and squamous cell carcinoma [47].

We used MEBOCOST to calculate mCCC scores for every metabolite-sensor partner between cell types in a scRNA-seq dataset. Following methods in recent reports [50, 63], we determined cell type colocalization score based on the Spearman correlation of spatial positions between sender and receiver cell types using ST data from the same tissue as the scRNA-seq data. Finally, we computed the Spearman correlation coefficient between mCCC communication scores and spatial colocalization scores between cell types for every metabolite and tissue type. For instance, the ADP-mediated mCCC between cell types positively correlated with spatial colocalization of the cells in the squamous cell carcinoma tissue (Fig. 3B). When considering all metabolites with significant mCCC, we observed an overall tendency for mCCC communication scores to correlate positively with the spatial colocalization scores between cell types (Fig. 3C). While the median correlation coefficient was around 0.2, ranging from 0.18 to 0.39, some metabolites negatively correlated with communication scores and spatial colocalization. This aligns with the observations of long-range cell-to-cell communications in recent ST studies [48, 62, 64]. We confirmed that these correlations were significantly higher than those for non-significant mCCC and not presented in the analyses using mock ST data of randomly shuffled spatial positions (Fig. 3C). These results verified that MEBOCOST is optimally designed to identify mCCC events, which appeared more likely to occur between spatially proximal cells, consistent with the established knowledge [48, 61, 62].

Next, we tested if the mCCC events detected by MEBOCOST were functional by analyzing a colorectal tumor scRNA-seq dataset [65, 66] in combination with a CRISPR screen dataset [67]. Applying MEBOCOST to the scRNA-seq data, we identified 31 mCCC events between the cancer and natural killer (NK) cells, involving 20 metabolites and 23 sensors (Supplementary Fig. S2A and B). In the CRISPR screen data, colorectal cancer cells transfected with genome-wide sgRNAs were cultured alone or co-cultured with NK cells [67]. We aimed to elucidate the function of mCCC genes by comparing their sgRNA counts in scenarios with and without NK cell co-culture. This approach will identify genes whose knockout (KO) results in either resistance or sensitivity of cancer cells to NK cell-mediated killing, evidenced by enriched sgRNAs and depleted sgRNAs, respectively (Fig. 3D). We observed that the genes involved in significant mCCC between cancer and NK cells exhibited a significantly higher rate of sgRNA depletion than sgRNA enrichment (Fig. 3E). As a control, this pattern was not observed for genes in non-significant mCCC. Moreover, compared to other enzymes and sensors, high RNA expression of enzyme and sensor genes in those detected mCCC events is associated with poorer patient survival in the TCGA colorectal cancer dataset, as suggested by a significantly higher patient hazard ratio (Fig. 3F).

It has been reported that immune cells can be hijacked by cancer cells and further fuel metabolites to drive oncogenic progression in the tumor microenvironment [68–70]. Meanwhile, cancer cells can reprogram immune response by releasing metabolites [71], such as gamma-Aminobutyric acid (GABA) [72, 73], lactate [74], and prostaglandin E2 [75–77]. Consistent with these reports, our findings indicated that colorectal cancer cells may evade NK cell-mediated killing via mCCC. For instance, GABA is a neurotransmitter but can be expressed by cancer cells to regulate cancer progression and tumor immunity [14, 72, 73]. In the colon tumors, MEBOCOST analysis revealed that GABA mediated the mCCC between colon cancer and NK cells through two sensors, namely, SLC6A6 and GABRG2 (Supplementary Fig. S2B). GABA enzyme genes were highly expressed in malignant cells compared to NK cells (Supplementary Fig. S2C). Interestingly, the CRISPR screen further revealed that ALDH9A1, a key enzyme that catalyzes gamma-aminobutyraldehyde to GABA [78], showed significant sgRNA depletion but not sgRNA enrichment in cancer cells cocultured with NK cells (Supplementary Fig. S2D and E). This suggested that GABA may help cancer cells escape NK killing. We hypothesized that GABA may be negatively associated with cancer patient survival. To test this hypothesis, survival analysis was performed using GABA enzyme and sensor gene expression in the TCGA colon cancer cohort. Interestingly, the high expression of GABA enzyme and sensor genes was significantly associated with poor patient survival (Supplementary Fig. S2F and G). This observation was consistent with the previous reports about the immunosuppressive roles of GABA through reducing the cytotoxicity and migration of NK cells [14, 72, 73]. These results implied that MEBOCOST is an effective tool for identifying functionally critical mCCC events.

### MEBOCOST outperformed general CCC methods in inferring mCCC

We compared the MEBOCOST to existing CCC methods, including the CellPhoneDB [79], scConnect [20], and NeuronChat [21], which cover CCC mediated by a few small molecules. Firstly, likely because CellPhoneDB and scConnect were designed mainly for analysis of protein ligand-receptor analysis, only 199 small molecule-receptor partners and eight neurotransmitter-receptor partners were covered, respectively. NeuronChat was explicitly intended for neurotransmitter-mediated neuron-neuron communications and includes 220 small molecule-target pairs. In contrast, MEBOCOST currently contains 440 metabolite-sensor partners, thus is at least 2-fold more comprehensive than CellPhoneDB, scConnect, and NeuronChat. Second, CellPhoneDB used the gene expression of the last enzyme in the biosynthesis pathway to represent a small molecule in calculating communication score. scConnect and NeuronChat used several vital enzymes in the biosynthesis and uptake pathways in their calculations. However, many metabolites or small molecules participate in complex metabolism, including production and consumption. Also, the efflux and influx rate can help define the secretion and uptake of a metabolite in mCCC. None of these tools for general CCC analysis took consumptive reactions, efflux, or influx of a metabolite into consideration when inferring mCCC. In contrast to these algorithms, MEBOCOST considered both the generation and consumption reactions of a metabolite and employed a single-cell FBA strategy to estimate the secretion and uptake of a metabolite (Supplementary Fig. S3A). Therefore, MEBOCOST advanced to these existing methods in the algorithm design for mCCC analysis.

Next, we analyzed ST and CRISPR screen datasets to compare the performance of mCCC inference using MEBOCOST, CellPhoneDB, scConnect, and NeuronChat. Zero mCCC events were detected by the NeuronChat in all six scRNA-seq samples analyzed in this study. The scConnect detected only six mCCC events in an SCC sample and zero mCCC events in the other five samples. These are probably due to the specific design of NeuronChat and scConnect for neuron-neuron communication mediated by neurotransmitters, which may not frequently happen in non-nervous tissues. We then focused on the quantitative comparison between CellPhoneDB and MEBOCOST. Firstly, we observed that MEBOCOST resulted in stronger correlations between communication and spatial colocalization scores than CellPhoneDB in six datasets, with three being significant (Supplementary Fig. S3B). MEBOCOST also detected more mCCC events, with more metabolites and sensors in those mCCC events (Supplementary Fig. S3C–E). Second, we compared the performance of MEBOCOST and CellPhoneDB based on the CRISPR screen data [67] of colorectal cancer cells with and without NK cell coculture. In this dataset, upon sgRNA-mediated gene KO, sgRNA depletion suggested an increased sensitivity of cancer cells to NK killing, while sgRNA enrichment suggested resistance effects. Although both gene sets in those mCCC events detected by MEBOCOST and CellPhoneDB showed a higher sgRNA depletion than sgRNA enrichment, MEBOCOST resulted in a more significant difference in the MAGeCK score between sgRNA depletion and enrichment (Supplementary Fig. S3F). This result indicated that the KO of genes in mCCC detected by MEBOCOST caused a more significant increase in cancer cell sensitivity to NK killing. Also, those genes of mCCC detected by MEBOCOST showed a trend towards a stronger association with poor patient survival than those of mCCC detected by CellPhoneDB (Supplementary Fig. S3G). These results suggested that MEBOCOST outperformed the CellPhoneDB in identifying functional mCCC events using scRNA-seq data.

### Simulation data demonstrated great scalability and robustness of MEBOCOST

To evaluate the scalability of MEBOCOST, we generated a scRNA-seq dataset of 33 470 cells from the stromal vascular fraction (SVF) of BAT in mice housing at cold temperature (5°C) for 2 days (Cold2) [39, 40]. We applied MEBOCOST on the original data and a series of down-sampled data with reduced total cell numbers to test the effect of the library size on the mCCC analysis. The result indicated that the detected mCCC number remained relatively stable even when the total cell number was down sampled to retain only 30% of the original total cells (Supplementary Fig. S4A). To evaluate the similarity of detected mCCC between down-sampled datasets and the original dataset, we introduced two measurements, including “recaptured over total detected” and “recaptured over originally detected.” In the “recaptured over total detected,” the numbers of mCCC detected in both the down-sampled and original datasets were divided by the number of mCCC detected in the down-sampled dataset. In the “recaptured over originally detected,” the numbers of communications detected in both down-sampled and original datasets were divided by the number of communications detected in the original dataset. We observed that both “recaptured over total detected” and “recaptured over original” ratios remained above 80% even when the number of total cells was reduced to 30% of the original total cell number (Supplementary Fig. S4B).

We next tested the tolerance of MEBOCOST to sequencing noise. We generated multiple simulated noisy datasets with varying noise rates by adding random reads to the Cold2 scRNA-seq data. We then applied MEBOCOST to these noisy datasets and tested the ability to recapture those communications detected in the original Cold2 scRNA-seq data. The results showed that detected communications reduced substantially when the noise read number increased from 1 to 6 folds of the original scRNA-seq read number (noise-to-signal ratio from 1 to 6) (Supplementary Fig. S4C). However, the ratio of recaptured over total detected communications increased substantially, whereas the ratio of recaptured over originally detected communications decreased (Supplementary Fig. S4D). These results suggest that MEBOCOST is unlikely to detect communications from noise. In datasets with a noise-to-signal ratio of less than 1, the number of detected communications remained stable (Supplementary Fig. S4C). Although the ratios of recaptured over total detected and recaptured over original detected decreased slightly in response to the moderate increase of the noise-to-signal ratio from 0.01 to 1, both measurements remained above 60% (Supplementary Fig. S4D). Therefore, the performance of MEBOCOST is resistant to a moderate noise-to-signal ratio lower than 1. Also, the number of detected communications fluctuated at different noise-to-signal ratios appeared moderate. It could be further decreased when the cutoff to define communications was set to be more stringent (Supplementary Fig. S4C). Altogether, the analysis based on subsampling and noise simulation strategies demonstrated that MEBOCOST is a robust algorithm for predicting mCCC from scRNA-seq data.

### MEBOCOST revealed mCCC changes associated with human obesity

**Figure 4.**
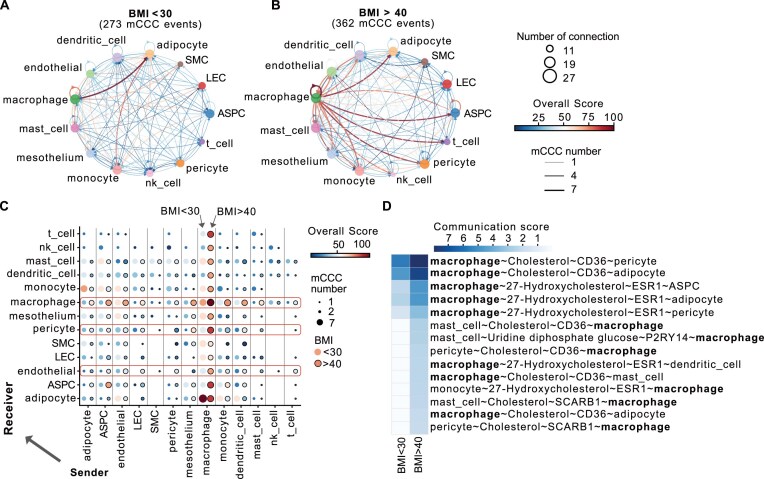
MEBOCOST revealed mCCC in WAT of patients during obesity. (**A**) and (**B**) network plots showing mCCC detected by MEBOCOST in scRNA-Seq data of WAT from BMI-low (BMI < 30, A) and BMI-high individuals (BMI > 40, B). Each dot represented a cell type. The dot size represented the number of communications of the cell type with the other cell types. Each arrow line represented the communication from a sender cell type to a receiver cell type. The line width indicated the number of metabolite-sensor communications between the sender and receiver cell types. Line color showed the overall communication strength calculated by the sum of -log10(FDR) of all mCCC events between the sender and receiver types. (**C**) The dot plot shows the communication scores between two cell types in BMI-high and BMI-low conditions. The X-axis showed sender cell types, and the Y-axis showed receiver cell types. Vertical blocks separated sender cell types across all receiver cell types. Within each block, the left column (dots without black circle) showed results of BMI-low individuals, and the right column (dots with black circle) showed results of BMI-high individuals. The dot size represents the number of communications. The dot color represents overall communication strength, calculated by the sum of -log10(FDR) of all mCCC events between the sender and receiver cell types. (**D**) A heatmap to show the highly increased macrophage-related mCCC (difference of communication scores between BMI-high and BMI-low individuals larger than 2). The color represented the original communication score. Each row showed a communication event.

We sought to employ MEBOCOST to explore mCCC dynamics in a disease process. We investigated mCCC changes during obesity using scRNA-seq data of human visceral WAT [43]. The data contained sixteen cell types in the WAT of individuals with different body mass index (BMI). These cell types included adipocyte, adipocyte stem and progenitor cell (ASPC), mesothelium, endothelial, lymphatic endothelial cell (LEC), pericyte, endometrium, smooth muscle cell (SMC), and eight immune cell types (Supplementary Fig. S5A-S5B). MEBOCOST identified 273 and 362 mCCC events in WAT of individuals with low BMI (BMI-low, BMI < 30) and individuals with high BMI (BMI-high, BMI > 40), respectively (Fig. 4A and B).

Intercellular communication in WAT is crucial in response to overnutrition [43, 80–82]. Chronic inflammation has been well-recognized during obesity [83]. Macrophage is one of the key cell types for promoting inflammation and inhibiting adipogenesis in obese WAT [84]. Consistent with this knowledge, we found that the number and strength of mCCC involving macrophages were greatly increased in BMI-high samples compared to BMI-low samples (Figs. 4A-C and Supplementary Fig. S5C). Specifically, macrophages received more mCCC from other cell types in BMI-high samples (54 mCCC events, 13.81% of total mCCC) than in BMI-low samples (30 mCCC events, 10.99% of total mCCC, Fig. 4C and Supplementary Fig. S5C). Meanwhile, macrophages send metabolites to other cell types with a stronger communication strength (Fig. 4B and C) in BMI-high samples than in BMI-low samples. Notably, we observed that cholesterol and 27-hydroxycholesterol (27-HC) were metabolites that mediate the most upregulated mCCC involving macrophage in the BMI-high samples (Fig. 4D). In addition to macrophages, cell types in the vascular system were also associated with increased mCCC, especially for those taking endothelial cells and pericytes as receivers (Fig. 4C and Supplementary Fig. S5C). Interestingly, the enhanced mCCC of vascular cells were primarily mediated by cholesterols (Supplementary Fig. S5D). These observations are consistent with the reports that the vascular system undergoes higher lipid transport and signaling during obesity [85–87]. Moreover, adipose tissue represents the most prominent body cholesterol reservoir [88]. The accumulation of cholesterol in adipose can increase up to 50% of the total free cholesterol in the body during obesity. The cholesterol efflux in adipocytes [89] and macrophages [90] can be up-regulated in the diet-induced mouse model. In line with this, Cummins *et al.* observed that the cholesterol level was significantly increased by 1.28-fold (FDR = 0.025) in epididymal fat (corresponding to visceral fat in humans) in the high-fat diet-induced obese mice [91]. Literature also reported an increase in other metabolites involved in obesity-associated mCCC in adipose tissues or plasma samples under obese conditions, including 27-hydroxycholesterol [92], choline [93], prostaglandin D2 [94], and uridine diphosphate glucose [95]. These reports supported the strong association between obesity and those metabolites predicted by MEBOCOST analysis.

### MEBOCOST recaptured known and identified new mCCC in mice brown adipose tissues

BAT specializes in dissipating chemical energy in the form of heat during thermogenesis. Cold exposure is an effective method for activating BAT [96–98] and promoting BAT remodeling, which triggers the proliferation and differentiation of adipocyte precursor cells [40, 99]. The process of brown adipocyte differentiation can be regulated by metabolites in BAT [100, 101]. To map the mCCC dynamics in the BAT in response to ambient temperature changes, we generated scRNA-seq data of BAT from mice housing at thermoneutrality (TN, 30°C for a week), room temperature (RT, 22°C), and cold temperature (5°C) for 2 days (Cold2) or 7 days (Cold7) [39, 40]. The data from those four conditions included 107,679 high-quality cells and 468 million reads from twenty cell types (Supplementary Fig. S6A-S6E). These cell types include adipocytes, adipocyte progenitor cells (APCs), vascular smooth muscle (VSM) cells, endothelial cells, Schwann cells, and different types of immune cells. Mature adipocytes are technically challenging to capture by single-cell isolation due to their large size, fragile nature, and high lipid contents. The captured adipocytes in our samples are those in the stromal vascular fraction (SVF) and are might be differentiating adipocytes with low lipid contents [40].

**Figure 5.**
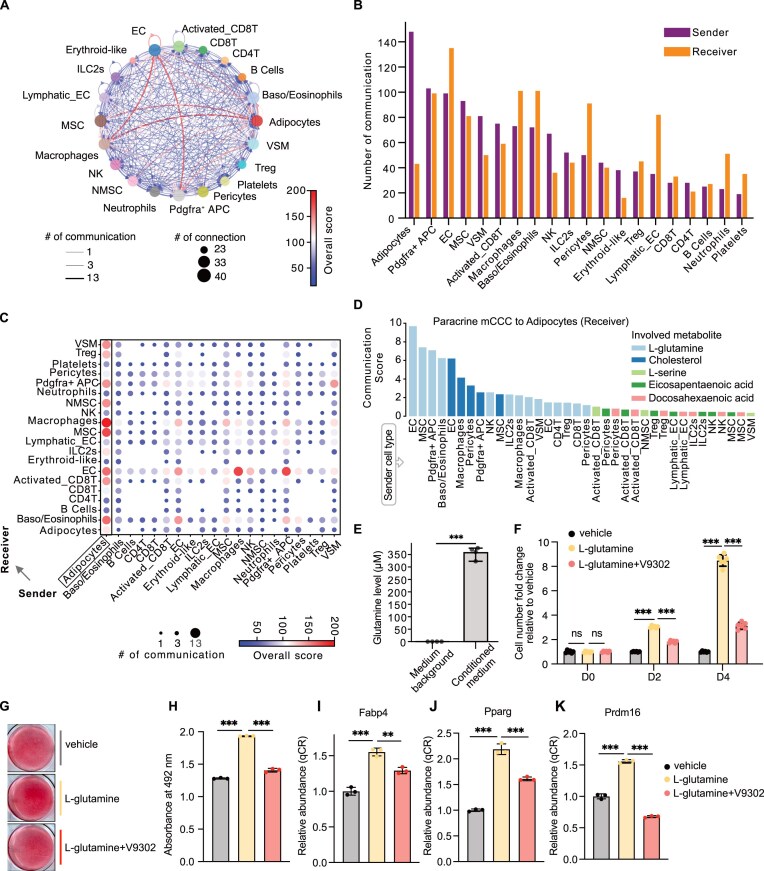
MEBOCOST recaptured known and revealed new mCCC in mouse BAT. (**A**) A network plot showing metabolite-sensor communications detected by MEBOCOST for the Cold2 BAT scRNA-Seq data. Dots, with different colors, represented the different cell types. The dot size represented the number of communications of the cell type with the other cell types. Each arrow line represented the communication from a sender cell type to a receiver cell type. The line width indicated the number of metabolite-sensor communications between the sender and receiver cell types. Line color showed the overall communication score calculated by the sum of -log10(FDR) of all metabolite-sensor communications between the sender and receiver types. (**B**) Bar plot to show the number of detected communications with each cell type as sender cells or receiver cells. (**C**) A dot plot showing the communications between each pair of cell types. The dot size indicated the number of metabolite-sensor communications between a pair of sender and receiver cell types. The dot's color represented the overall score of communications between the sender and receiver cell types. The black box highlighted adipocyte-associated communications. (**D**) A bar plot showing the communication scores for mCCC sending metabolites, including glutamine, to adipocytes in Cold2 BAT samples. The x-axis showed the sender cell types. The y-axis showed the communication score computed by MEBOCOST. The color labeled the different metabolites involved in the detected mCCC to adipocytes. (**E**) A bar plot shows the glutamine level in the conditioned medium of EC culture and the medium background (without EC culture). The y-axis is the glutamine concentration in μM. (**F**) Bar plots showing the proliferation of brown adipocyte progenitors treated by vehicle, L-glutamine, and a combination of L-glutamine and V9302 (L-glutamine transporter inhibitor). The y-axis is the fold change of cell number relative to that in the vehicle condition. (**G**)-(**H**) The Oil-Red O staining for the lipid accumulation for the adipocyte differentiation analysis. The G showed representative images of Oil-Red O staining for brown adipocytes cultured with differentiation medium supplemented with the vehicle, L-glutamine alone, or L-glutamine with V9302. The H showed the quantification of panel G’s Oil-Red O staining that measured the absorbance at 492 nm. I-K. The bar plots show the relative gene expression for mature brown adipocyte markers, *Fabp4*, *Pparg*, and *Prdm16*, in differentiated adipocytes, using a differentiation medium supplemented with the vehicle, L-glutamine, and a combination of L-glutamine and V9302. ** denotes *P*-value < 0.01, and *** denotes *P*-value < 0.001 of unpaired and two-tailed t-test. ns: non-significant.

We first analyzed the data of the Cold2 condition because the presence of differentiating adipocytes allowed us to interrogate the interactions that might facilitate brown adipocyte differentiation (Supplementary Fig. S6F). We detected 1,190 mCCC events in the Cold2 BAT for both paracrine and autocrine communications (Fig. 5A). Among all observed communications, those occurring from adipocytes to other cell types showed higher communication numbers and scores than those among other cell types (Fig. 5B and C). Meanwhile, adipocytes receive metabolites via paracrine and autocrine mCCC, respectively (Fig. 5A). Examples of autocrine metabolite-sensor partners include the L-glutamine-Slc1a5, L-glutamine-Slc38a2, eicosapentaenoic acid-Ffar4, and docosahexaenoic acid-Ffar4 (Supplementary Fig. S7A). Some of those metabolites, sensors, and metabolite-sensor partners have been reported to be involved in regulating the function of BAT. For example, omega-3 eicosapentaenoic acid (EPA) and docosahexaenoic acid (DHA) were essential in brown adipocyte differentiation and thermogenesis [102]. Jiyoung Kim and colleagues reported that EPA can potentiate brown adipocyte thermogenesis dependent on Ffar4 [103]. Glutamine was reported as a major source of *de novo* fatty acid synthesis in a brown adipocyte cell line [104], and the utilization of glutamine was reported to be enhanced in brown adipose tissue by acute cold exposure [105]. These observations demonstrated that MEBOCOST effectively recaptured known mCCC events in brown fat tissues.

In addition to autocrine, adipocytes received many paracrine mCCC from other cell types in the BAT. The most frequent sender cell types in those paracrine mCCC of adipocytes include the EC, MSC, pericytes, activated CD8T, VSM, non-NMSC, and Pdgfra^+^ APC (Fig. 5C and D). Among these sender cells, the communications between the vascular cells and adipocytes were well known to modulate adipocyte function [81]. For instance, using an EC-adipocyte co-culture system, Jennifer H Hammel and Evangelia Bellas showed that EC-adipocyte crosstalk improved the browning of white adipocytes [106]. MEBOCOST also identified some novel sender cell types in BAT. For example, activated CD8T cells, a subtype of CD8T cells expressing cytotoxic markers, were little known to communicate with adipocytes in BAT. Interestingly, 4 communications from activated CD8T cells to adipocytes were detected in Cold2 BAT, while only one communication was detected for naïve CD8T cells (Fig. 5C). Among all metabolite-sensor partners, we observed the highest communication scores of mCCC mediated by L-glutamine and Slc1a5, including those between EC and adipocytes, Pdgfra + APC and adipocytes, MSC and adipocytes, Baso/Eosinophils and adipocytes, etc. (Supplementary Fig. S7A). These observations support a hypothesis that adipocytes cross-talked frequently with other tissue-resident cells through metabolites in BAT.

To obtain insights into the potential function of those mCCC events detected in BAT, we analyzed the pairwise correlation between the RNA expression of genes in the mCCC events and enrichment scores of KEGG pathways across 629 bulk RNA-seq samples (Supplementary Fig. S8A). Enrichment scores of KEGG pathways in a bulk sample were calculated using single sample gene set enrichment analysis (GSEA) after excluding the mCCC genes in those pathways to avoid confounding effects on the enrichment score [107, 108]. It is worth noting that such a correlation analysis does not necessarily suggest a causal relationship between metabolite and pathway activation. Instead, this analysis may provide functional associations or insights into the detected mCCC in brown adipose tissue. Interestingly, the expression of mCCC genes exhibited strong correlations with pathway enrichment scores in two clusters of KEGG pathways, referring to “pathway cluster 1″ and “pathway cluster 2″ (Supplementary Fig. S8B). Word-cloud analysis [109] based on the pathway names of these two clusters indicated that “pathway cluster 1″ and “pathway cluster 2″ comprised metabolism-related and signaling-related pathways, respectively (Supplementary Fig. S8C and D).

Intriguingly, the metabolism-related pathways in “pathway cluster 1” include the “regulation of lipolysis in adipocytes,” a crucial process related to the thermogenesis of brown adipocytes [110] (Supplementary Fig. S8B). These pathways were associated with mCCC events mediated by essential metabolites, including L-glutamine, adenosine, heme, and cholesterol. Meanwhile, mCCC mediated by metabolites such as L-serine, D-mannose, and L-arginine showed a strong association with signaling-related pathways in “pathway cluster 2” (Supplementary Fig. S8B), including the “VEGF signaling pathway” and “TGF-beta signaling pathway.” Bagchi *et al.* have reported that the VEGF can promote brown adipocyte proliferation, and the loss of VEGF isoform impaired the BAT development [111]. Additionally, Tseng and colleagues discovered that bone morphogenetic protein 7 (BMP7), a gene of the TGF-beta signaling pathway, can promote brown adipocyte differentiation and thermogenesis *in vivo* and *vitro* [112]. Furthermore, the metabolite-sensor pair of L-glutamine and Slc1a5 was strongly correlated with pathway activities in “Pathway cluster 1.”. We analyzed an RNA-seq dataset in brown adipocytes with and without L-glutamine treatment [113]. The GSEA identified the up-regulated and down-regulated pathways. Interestingly, the positively correlated pathways with L-glutamine∼Slc1a5 (correlation coefficient > 0.3) tend to be enriched in the significantly up-regulated pathways in L-glutamine treatment (Supplementary Fig. S9A). The overlapped pathways include “Fatty acid metabolism,” “Regulation of lipolysis in adipocytes,” and other pathways that are crucial to brown adipose functions (Supplementary Fig. S9B), indicating the critical role of L-glutamine in brown adipose tissue. These observations implied a potential of resident cells within brown adipose tissue to collectively remodel the tissue and regulate essential functions of brown adipose tissue, including metabolism and signaling transduction.

### MEBOCOST revealed glutamine function in brown adipocyte differentiation

Several studies demonstrated that glutamine metabolism is likely associated with BAT thermogenesis [105, 113, 114]. Analysis of BAT scRNA-seq data from Cold2 mice revealed L-glutamine as a primary metabolite for mediating mCCC from multiple cell types to adipocytes, with EC showing a strong communication score (Figs. 5D). The glutamine-mediated mCCC can also be sensed by Pdgfra + APC (Supplementary Fig. S10A), a cell type known to proliferate and differentiate into mature brown adipocytes under cold exposure. Moreover, L-glutamine enzymes were highly expressed in a few BAT cell types, including EC, while the glutamine transporter Slc1a5 was enriched in brown adipocytes and Pdgfra + APC (Supplementary Fig. S10B). These findings motivated us to hypothesize that glutamine-mediated mCCC may play a role in regulating brown adipocyte differentiation.

To this end, we first tested if the L-glutamine can be secreted by the predicted sender cell types in our analysis, i.e. EC. Using the glutamine assay kit, we observed that the glutamine concentration was significantly higher in the conditioned medium of EC culture compared to the medium background (Fig. 5E), suggesting that EC secreted glutamine into the extracellular space. Next, we explored the impact of extracellular glutamine on APC proliferation and brown adipocyte differentiation, which are crucial steps to producing functional mature brown adipocytes [115]. Intriguingly, the glutamine treatment significantly increased APC proliferation compared to vehicle control (Fig. 5F). The brown adipocyte differentiation was also markedly enhanced upon the glutamine treatment. This was noted by a substantially higher level of lipid accumulation (Fig. 5G and H) and higher expressions for mature brown adipocyte markers (Fig. 5I–K), including *Fabp4*, *Pparg*, and *Prdm16*.

Furthermore, perturbation experiments were performed to verify the sensor proteins involved in L-glutamine-mediated mCCC to brown adipocytes. SLC1A5 and SLC38A2 are the two major transporters for L-glutamine-mediated mCCC events detected in brown adipocytes. We hypothesized that both SLC1A5 and SLC38A2 play critical roles in mediating L-glutamine mCCC to brown adipocytes, but each might compensate for the loss of the other when perturbed. We first knocked down SLC1A5 and SLC38A2 individually in the brown preadipocyte using the CRISPR/Cas9 technique and then differentiated them into mature brown adipocytes (Supplementary Fig. S11A and B). Consistent with our hypothesis, knocking down either SLC1A5 or SLC38A2 alone did not significantly affect the brown adipocyte differentiation compared to the control cells in the presence of L-glutamine (Supplementary Fig. S11C and D). Next, cells were treated with L-glutamine alongside with additional supplementation of V9302, a small molecular inhibitor initially identified to inhibit SLC1A5 but later found to also target SLC38A2 [116–119]. Notably, V9302 significantly reduced the effects of L-glutamine on APC proliferation (Fig. 5F), adipocyte differentiation (Fig. 5G and H), and mature adipocyte marker gene expression (Fig. 5I–K) compared to L-glutamine treatment alone. These results suggested that simultaneous inhibition of SLC1A5 and SLC38A2 attenuated the effects of L-glutamine on enhancing APC proliferation and brown adipocyte differentiation. Altogether, our experiments verified that cell types, i.e. EC, secrete glutamine that promotes the proliferation and differentiation of brown adipocyte progenitors through glutamine transporters SLC1A5 and SLC38A2.

### MEBOCOST detected temperature-sensitive mCCC in the thermogenesis process of BAT

**Figure 6.**
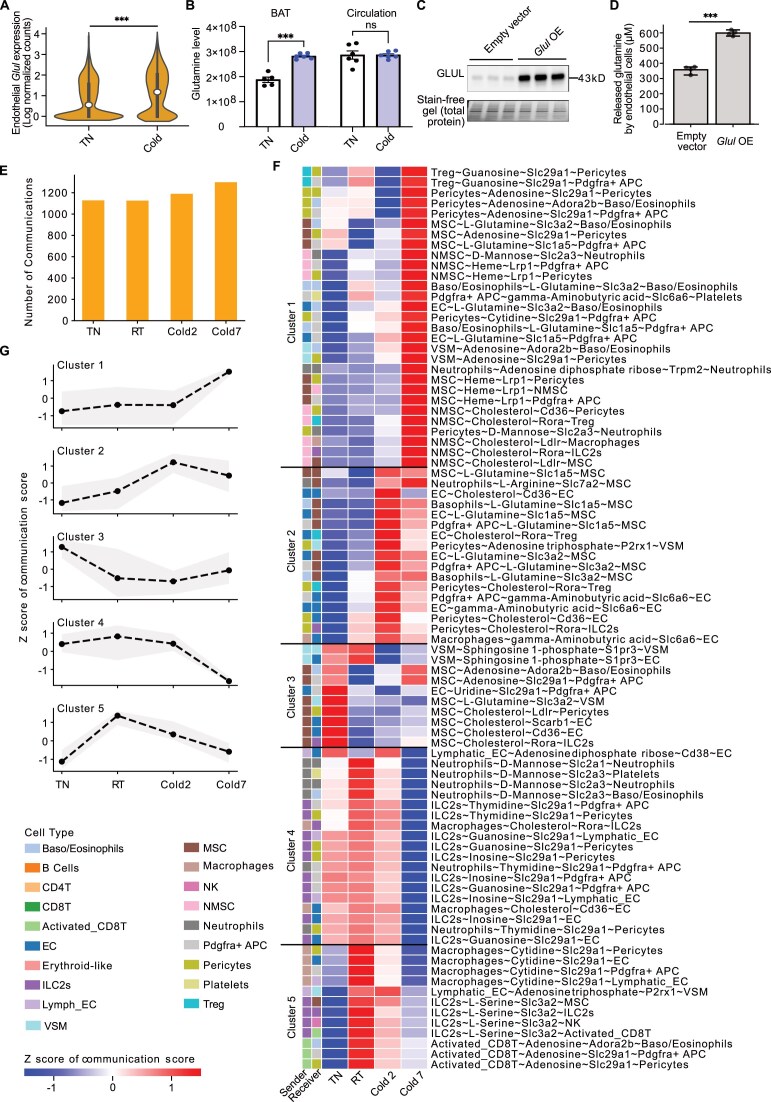
MEBOCOST revealed temperature-sensitive mCCC during mouse BAT thermogenesis. (**A**) The violin plot showing the Glul gene expression in EC of BAT scRNA-seq data in TN and Cold2 conditions. The plot includes 2173 and 1339 endothelial cells for cold and TN groups, respectively. (**B**) Bar plots showing the glutamine levels determined by mass spectrometry-based metabolomics for BAT and plasma between mice housing at cold temperature (5°C) and thermoneutrality. (**C**) The western-blot analysis showing the GLUL protein level in endothelial cells with empty vector and *Glul* overexpression. Three replicates were shown in each group. Total proteins from the stain-free gel were used as loading control. (**D**) A bar plot showing the released glutamine level by endothelial cells with the empty vector and *Glul* overexpression. The y-axis is the glutamine concentration in μM. ** denotes *P*-value < 0.01, and *** denotes *P*-value < 0.001 of two-tailed t-test. ns: non-significant. (**E**) A bar plot showing the total number of communications detected by MEBOCOST in BAT scRNA-seq data from mice under the TN (30°C for a week), RT, Cold2 (5°C for 2 days), and Cold7 (5°C for 7 days) conditions. (**F**) A heatmap showing detected mCCCs sensitive to the housing temperature of mice. Each row represented one mCCC event defined as a combination of a sender cell, metabolite, sensor protein, and receiver cell. Five mCCC clusters were identified using the K-means clustering method based on the mCCC scores. The values in the heatmap were the z-score of communication scores across conditions. The left sidebars labeled the sender and receiver cell types. (**G**) Line plots showed the pattern of changes in mCCC scores across different conditions for each of the five mCCC clusters. The dots showed z-scores of the mean communication scores of all mCCC events in the cluster, and the sallow showed the range of the communication z-scores from minimum to maximum.

By analyzing BAT scRNA-seq data, we observed a significant increase in the expression of *Glul*, a gene encoding glutamine synthetase, in ECs during cold exposure (Fig. 6A). Our mass spectrometry-based metabolomics analysis further revealed a significant increase in glutamine levels in BAT following cold exposure compared to thermoneutrality (Fig. 6B). However, such an increase was not observed in the circulation (Fig. 6B), suggesting a role for glutamine within BAT tissue independent of the circulation system during cold challenge. Additionally, glutamine secretion was significantly elevated due to higher expression levels of glutamine synthetase, achieved through GLUL overexpression in ECs (Fig. 6C and D). These observations led us to hypothesize that ambient temperatures may significantly regulate mCCC events in brown adipose tissue.

Next, we compared BAT mCCC among the four conditions, TN, RT, Cold2, and Cold7, to investigate temperature-regulated mCCC events. The number of detected mCCC was increased during thermogenesis in response to cold exposure, especially after the chronic cold exposure for 7 days (Fig. 6E), with 1128, 1126, 1190, and 1299 mCCC events in BAT of TN, RT, Cold2, and Cold7 conditions, respectively (Fig. 6E and Supplementary Table S2). Furthermore, the composition of mCCC in BAT was also changed dramatically. For instance, EC received the most significant number of mCCC from other cell types under the Cold2 conditions, an acute cold exposure (Fig. 5B). However, EC switched to the sender role in mCCC under TN, RT, and Cold7 conditions (Supplementary Fig. S12A–F). Moreover, MSC were the primary sender cells upon chronic cold exposure by 7 days, while EC, Pdgfra + APC, and adipocytes were the major senders in TN, RT, and Cold2, respectively (Fig. 5B and Supplementary Fig. S12A–F).

Furthermore, we identified three metabolite-sensor pairs that uniquely mediated mCCC in TN or RT but not in Cold2 and Cold7, including sphingosine-1-phosphate (S1P) ∼ S1pr1, S1P ∼ S1pr3, and GABA ∼ Slc6a13 (Supplementary Fig. S13). The correlation analysis was performed between metabolite-sensor gene expression and pathway activity in previously identified “pathway cluster 1.” The L-glutamine∼Slc1a5 was used as a control, showing a strong correlation with pathway activities in “pathway cluster 1.” However, all three TN- and RT-specific metabolite-sensor pairs showed significantly weaker or negative correlation with pathways in “pathway cluster 1” compared to L-glutamine∼Slc1a5 (Supplementary Fig. S13A-S13B). For example, those three metabolite-sensor pairs showed a negative correlation with the activity of pathway “regulation of lipolysis in adipocytes, opposite from L-glutamine∼Slc1a5 (Supplementary Fig. S13B). These results suggested that mCCC in mouse BAT was significantly regulated by ambient temperatures.

To identify the temperature-sensitive mCCC events, we calculated the index of dispersion (IOD) of communication scores for every mCCC event. Those mCCC displaying the most remarkable variation (the top 5% with IOD greater than 0.33) of communication scores were classified as temperature-sensitive mCCC. This analysis identified 87 such mCCC events, which were grouped into five clusters based on the pattern of communication scores (Fig. 6G). Notably, the mCCC in clusters 1 and 2 were those specifically enhanced by cold exposure compared to other conditions (Fig. 6F and G). Consistent with the increased glutamine in metabolomic mass spectrum analysis (Fig. 6B), L-glutamine-mediated mCCC events were enriched in clusters 1 and 2, including those from endothelial cells to APC. Additionally, NMSC and MSC were the primary sender cell types compared to other cell types and contributed to 48% (14 out of 29) of the mCCC in cluster 1. This result indicated that cold exposure may increase the secretion of metabolites from the peripheral nervous system and its effects on other adipose resident cells. The mCCC events within cluster 2 were characterized by a greater upregulation in the Cold2 condition compared to Cold7 (Fig. 6F and G), indicating that cold exposure remodels the mCCC in BAT differently in response to different levels of cold challenge. In contrast, the mCCC in clusters 3 and 4 were downregulated in the Cold7 condition relative to TN (Fig. 6F and G). However, cluster 5 includes 12 mCCC events downregulated by either thermoneutrality or cold exposure, suggesting that these mCCC may be sensitive to temperature changes (Fig. 6F and G). Altogether, MEBOCOST enabled the detection of condition-specific mCCC in BAT.

## Discussion

We developed a computational pipeline, MEBOCOST, to accurately identify mCCC events in scRNA-seq samples. To evaluate the performance of MEBOCOST, we used ST and CRISPR screen datasets to test the biological significance of mCCC detected in scRNA-seq data. For the evaluation based on ST datasets, we observed overall positive correlation coefficients, ranging from 0.18 to 0.39, between the mCCC communication scores defined by MEBOCOST using the scRNA-seq data and spatial colocalization scores calculated based on ST data (Fig. 3A–C, Supplementary Fig. S3B, and Supplementary Fig. S14), respectively. This observation suggested that the mCCC events identified by MEBOCOST are more likely to happen between spatially proximal cell types, consistent with the existing knowledge regarding CCC. It has been observed in recent ST analyses that some signaling molecules can mediate long-range cell-to-cell communications [48, 62, 64]. We indeed observed some metabolites that showed negative correlation coefficients between mCCC and spatial colocalization scores, meaning that those metabolites tend to mediate mCCC among spatially distal cell types. Using CRISPR screen data, we further validated that MEBOCOST can detect functional mCCC events in tumors. Specifically, deleting enzyme and sensor genes involved in mCCC in colorectal cancer cells increased the sensitivity of cancer cells to NK cell-mediated killing. Consistently, the expression of enzyme and sensor genes involved in those mCCC events was associated with poorer patient survival in the TCGA colorectal cancer dataset. These results indicated that cancer cells may suppress the NK cell function through those detected mCCC between cancer and NK cells in colorectal tumors.

We also demonstrated the usefulness of MEBOCOST in analyzing mCCC in adipose tissues. It has been reported that different cell types in adipose tissue cross talk extensively to form the adipocyte niche and regulate the function and development of adipose [120]. Since adipose is a metabolically active tissue, metabolites play important functions as signaling mediators [121]. MEBOCOST generated a comprehensive map of mCCC in brown and WATs, which will facilitate the investigation of cell type coherence in regulating the function and development of adipose tissue. We showed that MEBOCOST can detect condition-specific mCCC, which included applications to scRNA-seq data of BAT from mouse housing at different temperatures and WAT from patients with different BMI. As a robust stimulus to activate brown adipocytes, cold exposure remodels cellular composition and protein ligand-mediated intercellular communications in the tissue [39]. It has been known that the vascular cells and nerve terminals will be expanded to enable the maximal thermogenic activity of brown adipocytes upon cold exposure [122]. Consistently, we observed that endothelial cells, pericytes, VSM cells, and Schwann cells were involved in upregulated mCCC events in BAT upon cold exposure (clusters 1 and 2 in Fig. 5B and C). By analyzing WAT scRNA-seq data from patients with different BMI, MEBCOOST dissected the dynamic change of mCCC during obesity. Specifically, we observed that macrophages were among the top cell types involved in those upregulated mCCC events in BMI-high WAT (BMI > 40) compared to BMI-low samples (BMI < 30). Cholesterol and 12-hydroxycholesterol were the most frequently detected metabolites mediating BMI-high specific macrophage-associated mCCC. It has been reported that cholesterol transport was enriched in adipose macrophages in obese mice and was associated with the regulation of macrophage chemotaxis, one of the key contributors to inflammation [123, 124]. Our findings regarding macrophage-mediated mCCC through cholesterol provide insights into how the intercellular transport of cholesterol might regulate macrophages and tissue remodeling in white adipose in obesity. By comparing MEBOCOST metabolites to other metabolites, we showed that metabolites in MEBOCOST database tend to exhibit higher efflux and influx activity (Supplementary Fig. S15), consist with their role as extracellular metabolites involved in intercellular communication. Moreover, our results showed that mCCC detection in MEBOCOST was not strongly associated with cell population size, suggesting robustness against the effect of cell cluster size on mCCC analysis (Supplementary Fig. S16). Altogether, MEBOCOST, a versatile and user-friendly Python package, enables mCCC analysis using scRNA-seq data across various human and mouse samples under various conditions.

MEBOCOST is a computational method specifically dedicated to mCCC analysis, allowing us to tailor the algorithm design for features different from protein-mediated CCC. By continuing to develop new algorithms and functions, the capability of MEBOCOST will be further enhanced through multiple significant aspects in the future. The number of extracellular metabolites can be substantially increased by integrating more metabolite resources such as HMDB, Recon2, Metabolic Atlas, etc. The text-mining pipeline to improve the recognition of metabolite-sensor partners will also be further optimized. For example, we will enable the pipeline to use synonymous metabolites and sensors to parse the metabolite-sensor partners from the literature. Also, integrating more information in communication prediction may increase the algorithm's performance due to the complicated biological mechanism of cellular communication. First, cell surface transporters or receptors for metabolites might function by a protein complex. For example, SLC7A11 is a cell surface transporter for cysteine curated in our database, but SLC3A2 is an extra need for forming the SLC7A11-mediated mCCC [125]. Therefore, improving MEBOCOST by incorporating the protein complex information of sensors into the communication score calculation might be helpful. Additionally, MEBOCOST infers potential mCCC using single-cell transcriptomics data without considering the spatial proximity of the cells. Recently, ST has been successfully utilized to improve the prediction of protein-based cell–cell interaction [5, 7, 8, 79], demonstrating the informative role of spatial information in CCC detection. Although a robust performance has been observed in the current MEBOCOST, it has the potential to provide a more comprehensive view of mCCC by combining the spatial distribution of the cells into consideration. The downstream effects of a mCCC can be complicated in the receiver cells. The cell surface receptor-mediated mCCC may trigger the activation of signaling pathways to regulate biological processes in receiver cells [52]. The cell surface transporter-mediated mCCC allows metabolites to travel into receiver cells and participate in various biological processes, including metabolism [126], signaling transduction [127], epigenetic regulation [128, 129], etc. The nuclear receptor-mediated mCCC also exerts significant effects on the receiver cells, as nuclear receptors activated by metabolite ligands can bind to chromatin and regulate the transcription activity of many genes [59, 130]. Future works can be conducted to elucidate the downstream effects of mCCC, which would offer valuable insights into the functional role of those extracellular metabolites in tissue niches.

## Supplementary Material

gkaf569_Supplemental_Files

## Data Availability

Python implemented all algorithms in MEBOCOST. The collected enzyme and sensor genes of extracellular metabolites, the source code of MEBOCOST, and detailed instructions for usage are available at Figshare (https://doi.org/10.6084/m9.figshare.28291367.v1) and GitHub (https://github.com/kaifuchenlab/MEBOCOST). All the code for generating plots for this study can be found at Figshare (https://doi.org/10.6084/m9.figshare.28291850.v1) and GitHub (https://github.com/kaifuchenlab/mebocost_paper_scripts). The single-cell RNA-seq data of mouse brown adipose tissue is available at NCBI GEO under the accession number GSE160585. The summary table of public datasets used in this study can be found in Supplementary Table S3.
