# Supplementary material for: MEBOCOST maps metabolite-mediated intercellular communications using single-cell RNA-seq
Source: Nucleic Acids Res. 2025 Jun 26;53(12):gkaf569. doi: 10.1093/nar/gkaf569 (PMC12199156; doi:10.1093/nar/gkaf569)
Supplement: gkaf569_Supplemental_Files [file gkaf569_supplemental_files.zip › Supplementary_Note_figure_method.v2.pdf]

Supplementary Note:

Supplementary Methods

Software and package version

Cellranger 6.1.2, scFEA 1.1.2, COMPASS (https://github.com/YosefLab/Compass, downloaded on October 19th, 2021), Python 3.8, and python packages including pandas 1.4.1, scipy 1.8.0, scanpy 1.8.2, matplotlib 3.5.1, seaborn 0.11.2, adjustText 0.7.3, network 2.7, jupyter 1.0.0, MEBOCOST v1.0.2, wordcloud 1.9.2. CellPhoneDBv4, R packages include GSVA_1.40.1.

Collection of extracellular metabolites and related enzyme genes

scFEA and COMPASS are two software for metabolic flux analysis in scRNA-seq data. The metabolites covered by the two software were analyzed in this manuscript. Specifically, the metabolite names and their annotation information in scFEA were downloaded from https://github.com/changwn/scFEA/blob/master/data/Human_M168_information.symbols.csv. The metabolite name and annotation in COMPASS were downloaded from https://github.com/YosefLab/Compass/blob/master/compass/Resources/Recon2_export/met_md.csv. We standardized the collected metabolite names from scFEA and COMPASS into the standard annotation provided by the Human Metabolome Database (HMDB; https://hmdb.ca/). To do this, we focused on the metabolites for which the HMDB accession number was known. HMDB accession numbers of metabolites can be directly accessed in COMPASS, while only KEGG accessions were provided for scFEA metabolites. To map those scFEA metabolites into HMDB annotation, a parser script was developed to convert KEGG compound accession number into HMDB accession number. Taking Acetyl-CoA as an example, C00024 is the KEGG compound accession number, and the KEGG page of C00024 is at https://www.genome.jp/entry/C00024. The related annotation in HMDB for Acetyl-CoA can be further found by hyperlinks at the “All links” session. In this specific case, the HMDB accession number for Acetyl-CoA can be collected from https://www.genome.jp/dbget-bin/get_linkdb?-t+hmdb+cpd:C00024. We applied the strategy for all the metabolites from scFEA to find their HMDB accession numbers. For metabolites successfully mapped to HMDB, biological location annotations, including cellular and biospecimen locations, were extracted. To focus on potential intercellular signaling metabolites, the metabolites in “extracellular space,” “blood,” or “cerebrospinal fluid” were retained and named as “extracellular metabolites.” Meanwhile, other basic annotation information for those metabolites was also collected, such as synonyms, metabolite class, and protein associations.

We focused on the extracellular metabolites collected by the abovementioned procedures to collect a list of metabolite-enzyme associations. A parser script was explicitly developed to collect the reaction and related enzymes of extracellular metabolites in HMDB. Briefly, the webpage of metabolite in HMDB was visited based on the given HMDB accession number, and the annotation in the “Enzymes” sessions was collected. For example, the annotation page of D-Lactic acid can be retrieved by https://hmdb.ca/metabolites/HMDB0001311 where HMDB0000171 is the HDMB accession number. In the “Enzymes” tab, the reactions such as “S-Lactoylglutathione + Water → Glutathione + D-Lactic acid” and the related gene name (e.g. HAGH) were collected. Only complete reactions containing metabolite names for substrates and products were included in the collection. Such procedures produce a list of metabolic reactions and the corresponding enzyme gene names and metabolite names, which will be used in the MEBOCOST analysis.

Collection of metabolite-sensor partners

The detection of metabolite-based intercellular communications relied on the prior knowledge of metabolite-sensor partners. The sensor proteins in this study mainly include three types, namely, cell surface transporter, cell surface receptor, and nuclear receptors. A workflow combining computational text-mining and manual collection was developed to collect known pairs of metabolite-sensor partners.

The text mining needed a list of metabolites and sensor gene names. For metabolites, we focused on the extracellular metabolites. We obtained cell surface receptors from two sources, as one category of sensor proteins. Firstly, we downloaded a list of receptors from NicheNet(1), a protein ligand-receptor communication analysis R package, by taking the “to” genes in the file at https://zenodo.org/record/3260758/files/lr_network.rds. Secondly, we collected receptors by the keyword “receptor” from the UniProt database(2) at https://www.uniprot.org/uniprot/?query=name%3Areceptor+reviewed%3Ayes+organism%3A%22Homo+sapiens+%28Human%29+%5B9606%5D%22&sort=score. The receptors from these two sources were united and further filtered by focusing on cell membrane proteins based on the cellular location annotation in the UniProt database. For cell surface transporters as the second category of sensor proteins, we noticed that the Transporter Classification Database (TCDB)(3-7) contains a comprehensive list of transporter proteins. Thus, transporter gene names were collected from TCDB at https://www.tcdb.org/hgnc_explore.php. For nuclear

receptors as the third category of sensor proteins, the Nuclear Receptor Signaling Atlas (NURSA)(8) in dkNET project at https://dknet.org/data/source/nif-0000-03208-6/search was used for collecting known nuclear receptor names. After having the names of metabolites and sensors, text-mining was performed on PubMed abstracts. Firstly, 711,272 PubMed abstracts were obtained from https://eutils.ncbi.nlm.nih.gov/entrez/eutils/esearch.fcgi?db=pubmed&term=%28metabolism%5BTitle%2FAbstract%5D%29&retmax=15000000. We started from those abstracts as they were related to metabolism. Such a strategy helped us to narrow down to a subset of abstracts, thus reducing the time of text-mining compared to going through all the abstracts in PubMed. Secondly, the context of publication titles, MeSH words, and abstracts was downloaded for those PMID. Thirdly, potential combinations of metabolite and sensor names were checked in each collected context of publication titles, MeSH words, and abstracts. The PMID, metabolite names, and sensor names were recorded if metabolite and sensor names were co-mentioned in the same sentence in the context of publication titles, MeSH words, or abstracts. Next, the collected pairs of metabolite-sensor partners and the PMID and evidence in the context of publication title, MeSH words, or abstracts were subjected to manual curation.

Besides the text mining, we manually collected metabolite-sensor partners from well-known databases such as the HMDB(9-12), Recon2(13), GPCRdb(14,15), Wikipedia, and GeneCards(16). For HMDB, we collected metabolite and transporter pairs if the metabolite-associated protein was annotated as a transporter and the protein belongs to a type of cell membrane protein. For Recon2, since metabolites were further annotated by cellular localization, such as [e] for extracellular and [c] for cytosol, we specifically collected the reaction-associated genes if the reaction happened by transporting the same metabolite from extracellular [e] into the cytosol [c]. However, HMDB(9-12) and Recon2(13) did not have cell surface and nuclear receptors. Therefore, only metabolite and cell surface transporter pairs were mainly focused in these two databases. We also collected metabolite and cell surface receptor partners from GPCRdb(15,17), a database for G protein-coupled receptors (GPCR) annotation and their ligands. The metabolite ligands of the GPCR proteins were collected from the webpage at https://gpcrdb.org/ligand/statistics. Additionally, one pair of metabolite-sensor partners, pyruvic acid and SLC16A11, was collected from the Gene Card database by reading the description of *SLC1611* at https://www.genecards.org/cgi-bin/carddisp.pl?gene=SLC16A11. Next, we manually collected metabolite and nuclear receptor partners from a Wikipedia page at https://en.wikipedia.org/wiki/Nuclear_receptor#Ligands.

All the metabolite-sensor partners were manually curated by at least three curators separately. The criteria of the manual curation were below. For transporters, the metabolite-sensor pair will be collected if any abstracts explicitly mentioned roles of transporter, uptake, or influx for a metabolite through a transporter. For example: “participation of cd36 in membrane calcium influx”, “ABCA1 mediates high-affinity uptake of 25-hydroxycholesterol”, “L-arginine transporter gene SLC7A1”, “The proteins identified so far are cholesterol transporters such as SR-BI (scavenger receptor class B type I), CD36 (cluster determinant 36), NPC1L1 (Niemann-Pick C1-like 1) or ABCA1 (ATP-Binding Cassette A1)”. For receptors, the metabolite-sensor pair will be collected if any abstracts explicitly mentioned roles of binding, activation, or agonist for a metabolite through a receptor. For example: “GPR31 is a purported G-protein-coupled receptor for 12-HETE”, “Estrogen receptor antagonist 2-hydroxyestradiol”, “the cholesterol metabolite 25-hydroxycholesterol activates estrogen receptor”, “AMP is an ADORA1 agonist”, “TRPM2 is activated by adenosine diphosphate ribose and hydrogen peroxide”, etc. For those collected from RECON2 and HMDB database, the metabolite and transporters will be collected if any metabolic reactions from extracellular space to cytoplasm included that metabolite of interest. The criteria in the literature collections also applied on metabolite-sensor pair collections in the GPCRdb, WikiPedia, and Gene Card databases. Notably, the species and cell type were unrestricted during the computational text-mining and manual collection, although the metabolite enzyme and sensor names were primarily obtained from databases for humans. The metabolite enzyme genes and metabolite sensor genes were mapped to mouse genes based on the homology between human and mouse to generate a collection of metabolite-sensor partners for mouse.

Aggregating enzyme expression of a metabolite in a cell based on scRNA-seq data

When MEBOCOST performs initial filtering of metabolites based on their enzyme expression levels, the expression of enzymes is aggregated to aggressively filter out metabolite that is unlikely to be produced in a cell. It has been noticed that many metabolites participate in more than one reaction, and some reactions take the metabolites of interest as substrates. In contrast, other reactions may take those metabolites as products. We reasoned that those two types of metabolic reactions should dynamically influence the level of a given metabolite in a cell. The expression of the enzymes in generating reactions constrains the accumulation of those metabolites. However, the enzyme expression in the consuming reactions constrains the depletion of given metabolites because such metabolic reactions will convert those metabolites

into other ones. Therefore, we aggregated metabolite enzyme expression by calculating the average expression of enzymes involved in metabolite-generating reactions and subtracting the average expression of enzymes that utilize the metabolite as a substrate. The calculation based on enzyme gene expression was formulated as follows:

$$M = \frac{1}{l_p} \sum_{i=1; i \in P} x_i - \frac{1}{l_s} \sum_{j=1; j \in S} x_j$$

where M is the aggregated enzyme expression of a given metabolite in a cell. P is a set of enzyme genes that are associated with the accumulation of the metabolite. i is the ith enzyme gene in the P. l_p is the total number of enzyme genes in the P. In addition, S is a set of enzyme genes associated with metabolite depletion. j is the jth enzyme gene in the S. l_s is the total number of enzyme genes in the S. x_i and x_j are expression levels for enzyme i and j in a cell, respectively. It is important to note that this is a qualitative filtering of metabolites based on the enzyme expression and should not be recognized as a quantitative estimation of metabolite abundance, as the association between enzyme expression and metabolite production could be nonlinear.

Identification of significant co-expression of metabolite enzyme and sensor

Given the aggregated enzyme expression of metabolites and the prior knowledge of metabolite-sensor partners, MEBOCOST computed an enzyme-sensor co-expression score for each metabolite-sensor partner between each pair of cell types. For a pair of cell types i and j, and for a metabolite m and its sensor s, μ_m^i denotes the mean enzyme expression in cell type i, and μ_s^j denotes the mean sensor expression in cell type j. An enzyme-sensor co-expression potential S_c was computed as:

$$S_c = \mu_m^i * \mu_s^j$$

To evaluate the statistical significance of S_c, we performed permutation testing(18) (one-tailed) by shuffling cell labels across all cells of the scRNA-seq data. The same method was applied for each metabolite-sensor partner between each pair of cell types to calculate the enzyme-sensor co-expression score for each set of shuffled scRNA-seq data. We repeated this procedure 1,000 times to compute 1,000 S_c as a statistical *null* distribution for each metabolite-sensor partner between each pair of cell types. Based on the *null* distribution, a *p*-value was computed. The *p* values of all S_c scores for a dataset were subjected to a false discovery rate (FDR) correction by the Benjamini-Hochberg method(19). Furthermore, MEBOCOST provides three parameters, including “cutoff_exp”, “cutoff_met”, and “cutoff_prop”, for users to define

expressed metabolite enzymes and sensors in cell populations. “cutoff_exp” and “cutoff_met” were cutoffs to define expressed enzyme and sensor in each cell, respectively. “cutoff_prop” was a cutoff to define the proportion of cells that expressed enzymes of a metabolite and a sensor in a cell group (e.g., cell type). By default, “cutoff_exp” and “cutoff_met” were determined by taking the 25th percentile value of all sensor expression and all aggregated enzyme expression in all cells, respectively. The default “cutoff_prop” was set to 0.15, which means that at least 15% of the total cells in the cell group expressed the enzyme and sensor. The *p*-value and FDR of any communications with values lower than the three cutoffs will be converted to 1.

Calculation of communication scores

The flux balance analysis (FBA) is a well-established statistical method for calculating the flow of metabolites through the metabolic networks in the genome-scale metabolic models (GEM)(20). Integrating the FBA with gene expression data allows for investigating sample-specific metabolic flux(21,22), including metabolite secretion and uptake, which are crucial for an mCCC process. To constrain the mCCC events identified based on enzyme-sensor co-expression analysis, MEBOCOST integrated FBA to calculate efflux and influx rates of metabolites, determining their secretion and uptake potential in each cell population. It remains challenging to model the flux between two cell types without spatial information. However, several tools have been published to model the metabolic flux for each cell type or single cells, including scFEA, COMPASS, and scFBA (21,23,24). Their inferred metabolic fluxes include those metabolite effluxes from cells to the extracellular spaces, referring to the metabolite secretion process in mCCC. Metabolite influx from extracellular space to receiver cells was also included, referring to the metabolite uptake process. We hypothesized that adding the metabolite efflux and influx into consideration would improve the mCCC analysis’s performance. Because this integration with FBA will complement the enzyme and sensor co-expression analysis to further evaluate the secretion and uptake process. As it is challenging to model the metabolite flux between two cell types directly, we applied constraints on efflux rates for sender cells with extracellular space and constraints on influx rates for receiver cells with extracellular space, respectively. These two constraints can filter out low secretion and uptake metabolites in sender and receiver cells.

Users can choose to integrate FBA results from any tools of their interest that compute the metabolite efflux and influx rates, such as COMPASS, scFEA, etc. In this manuscript, COMPASS was implemented by taking the averaged gene expression matrix for cell

populations as input, then integrated all metabolic reactions in the RECON2 to compute the flux rates for each reaction including extracellular reactions. The results, stored in ‘secretion.tsv’ and ‘uptake.tsv’ files, form two metabolite-by-cell type matrices representing COMPASS scores for efflux and influx across all metabolites and cell types. To integrate gene expression and flux rates optimally, we tested the performance of five different methods for calculating mCCC scores, including “flux & sensor expression” for scoring mCCC scores by multiplying efflux/influx with sensor gene expression between sender and receiver cell types, “significant co-expression of enzyme and sensor” for significant co-expression of metabolite enzymes and sensors, “sig_coexpr + flux cutoff” for applying efflux/influx thresholds to constrain cell type pairs with significant enzyme-sensor co-expression, “sig_coexpr + flux * sensor expression” for scoring mCCC scores by multiplying efflux/influx rates with sensor gene expression values among cell type pairs with significant enzyme-sensor co-expression, “sig_coexpr + flux * co-expression of enzyme and sensor” for scoring mCCC scores by multiplying efflux/influx rates with enzyme-sensor co-expression values among cell type pairs with significant enzyme-sensor co-expression.

Since CCC events generally tend to happen between spatially proximal cells(25-27), we employed the correlation between mCCC scores and colocalization scores of cell types to evaluate the performance of the five methods. We applied the five methods on scRNA-seq samples from heart(28), intestine(29), pancreatic tumor(30), and squamous cell carcinoma(31). Following methods in recent reports(32,33), we computed the cell type colocalization score based on the Spearman correlation of spatial positions between sender and receiver cell types using spatial transcriptomics data from the same tissue as the scRNA-seq data. Next, we computed the Spearman correlation coefficient between mCCC scores and spatial colocalization scores between cell types for every metabolite and tissue type. The correlations of mCCC score and colocalization were shown in a violin plot (**Supplementary Figure 14A**), and the numbers of mCCC events for each sample were shown in a bar plot (**Supplementary Figure 14B**). The results showed that methods of “sig_coexpr + flux cutoff” and “significant co-expression of enzyme and sensor” significantly outperformed “flux & sensor expression”, suggesting that metabolite enzyme-sensor co-expression between sender and receiver cell groups is needed in mCCC analysis in addition to metabolite efflux/influx rates. However, a threshold to qualitatively define active metabolite efflux and influx events performed better than multiplying flux rate with gene expression in recognizing spatially colocalized mCCC cell type pairs. This was confirmed by higher median correlation values of mCCC and colocalization

scores in method of “sig_coexpr + flux cutoff” than methods of “sig_coexpr + flux * sensor expression” and “sig_coexpr + flux * co-expression of enzyme and sensor”. Additionally, the method of “sig_coexpr + flux cutoff” identified the lowest number of mCCC events across samples, indicating that the method with a qualitative constraint step for fluxes provides the most conservative inference than other methods. Altogether, we used “sig_coexpr + flux cutoff” method to idenfy the final mCCC events and compute the communication scores.

MEBOCOST then identifies highly confident mCCC events by applying these efflux and influx matrices to cell group pairs with significantly co-expressed enzymes and sensors. A cell group is considered a sender if its metabolite efflux score exceeds a certain threshold and, similarly, a receiver if the influx score surpasses this threshold. By default, MEBOCOST sets these thresholds at the 25th percentile of all metabolite scores across cell types. The idea of default thresholds of efflux and influx was to filter out those slowest fluxing metabolites across cell types. We choose the first quartile, namely the 25th percentile, to define those slowest fluxes based on several considerations. First, the efflux/influx score reflects the flux rate. A higher score means a faster flux, a lower score means a slower flux but not non-fluxing. Second, all metabolites in the MEBOCOST database were collected due to their roles in efflux or influx so they tend to have higher efflux and influx activity than other metabolites (**Supplementary Figure 15**). Many zero flux rates were observed for other metabolites but not for those metabolites in MEBOCOST, as shown in the **Supplementary Figure 15A and C**. Third, the default efflux and influx threshold for the MEBOCOST metabolites usually refer to a higher percentile (nearly the median) among all other metabolites (**Supplementary Figure 15B-D**). Also, users can decide any efflux and influx thresholds based on the distribution of efflux and influx scores for their own data.

The final communication score for each mCCC event between a pair of cell groups and a metabolite-sensor pair is determined by the enzyme-sensor co-expression score S_c, provided both efflux and influx scores meet their respective thresholds; otherwise, it will be set to zero. Notably, the influx constraint for receivers is only applied to mCCC events mediated by cell surface transporters and nuclear receptor sensors, which require metabolite entry into receiver cells. However, this influx constraint is not applied for mCCC events mediated by cell surface receptors, where metabolite entry into cells is not required. Thus, the final communication score for mCCC events mediated by cell surface transporters or nuclear receptors is formulated as follows:

$$S_f = \begin{cases} S_c, & v > c, v' > c' \\ 0, & v \leq c, v' \geq c' \\ 0, & v \geq c, v' \leq c' \\ 0, & v \leq c, v' \leq c' \end{cases}$$

The final communication score for a mCCC that mediated by cell surface receptors can be formulated as follows:

$$S_f = \begin{cases} S_c, & v > c \\ 0, & v \leq c \end{cases}$$

In these two equations, the S_f is the final communication score for a mCCC event, the S_c is the defined enzyme-sensor co-expression score in the last step, the v and v' are the efflux score computed by COMPASS software in sender and receiver cell groups, respectively. The c and c' are the defined thresholds for efflux and influx scores, respectively.

10x Genomics single-cell RNA sequencing

The single-cell RNA-seq data of mouse brown adipose tissue from TN (30 °C for 7 days), RT (22 °C), Cold2 (5 °C for 2 days), and Cold7 (5 °C for 7 days) was generated by 10X Genomics platform(34,35). Nine-week-old male C57BL/6J mice (stock no. 000664) purchased from Jackson Laboratory were used. Four mice were used in each condition. Brown adipose tissues from the interscapular region were dissected. Collected tissues were minced and subjected to digestion and dissociation. The top layer of adipocyte and supernatant in dissociated materials were removed. Note that adipocytes in the top layer were likely mature and fragile due to the enrichment of multilocular lipid droplets in the cells. Next, the stromal vascular fraction (SVF) was collected, resuspended, and filtered through a 100-μm cell strainer. The red blood cells were lysed by sterile ammonium–chloride–potassium lysis buffer (ACK Lysing Buffer, Lonza). Dead cells were further removed using the Dead Cell Removal kit (Miltenyi Biotec). The detailed protocol of SVF isolation from brown adipose tissue for scRNA-seq can be found at protocols.io (https://www.protocols.io/view/isolation-of-stromal-vascular-fraction-svf-from-mo-81wgb7y2ovpk/v1). For single-cell RNA sequencing, a 10x Genomics Chromium Controller instrument (10x Genomics) was used to generate single-cell droplets. The scRNA-seq libraries were prepared by the Chromium Single Cell 3′ version 3 Reagent kit following the 10x Genomics protocol. NovaSeq S2 flow cell (Illumina, 100 cycles) was used for RNA library sequencing.

Single-cell RNA-seq data processing

For the mouse BAT scRNA-seq data, cellranger was applied to map the raw sequence to the mouse reference genome (mm10) and obtain the read count over each gene, with r1-length parameter set to 26 and other parameters were the default values. Next, the data processing, including data normalization, dimension reduction, clustering, and visualization of gene expression, was performed using Scanpy(36). Cells were filtered to have at least 800 UMIs and 400 detected genes. Genes were filtered to be at least detected in 10 cells. To reduce the doublet effect, cells were removed if the total UMIs were more than 50,000 or the number of detected genes was greater than 7,500. The number of nearest neighbors was set to 10 in the Scanpy "find neighbor" function. The top 40 principal components were included in clustering and UMAP analysis(37). The visualization of the clusters was performed by the UMAP method. Cell annotation was done based on the cell type marker genes collected from PanglaoDB(38) at https://panglaodb.se/markers.html.

Cell type deconvolution and calculation of cell type colocalization of 10x Visium spatial transcriptomics

We acquired the read count matrix for spatial transcriptomics (ST) data on the Visium platform from Liu et al(27). Visium ST technology maps transcriptome in spots in tissue sections in tissue section spots, and deconvolution analysis can reveal the cell type composition within each spot. We used STRIDE(39) to deconvolute the proportion of cell types in every spot of the ST sample by integrating the scRNA-seq data with well-annotated cell types as references. We computed the colocalization score for any two cell types as the Spearman correlation of their proportions across all spots, following methodologies described in prior studies(32). To assess the alignment between detected mCCC and spatial colocalization, we calculated the Spearman correlation between communication and colocalization scores for each cell type pair. This analysis covered all metabolites in significantly detected mCCC cell type pairs. We averaged the communication scores of metabolite-mediated mCCC through multiple sensors between a cell-type pair. Additionally, we generated random spatial datasets by shuffling the spatial positions on the cell-type proportion matrix, serving as a background control for the correlation analysis. The results of this analysis were used in generating **Figure 3B-C** and **Supplementary Figure 3B**.

Analysis of public scRNA-seq data, CRISPR screen data, and patient survival data

Several public scRNA-seq datasets were used in this study. For the WAT scRNA-seq with different body masses for **Figure 4** and **Supplementary Figure 5**, the normalized gene

expression data and cell type annotation were downloaded from the Single Cell Portal with accession number SCP1376. For scRNA-seq data used in **Figure 3B-C** and **Supplementary Figure 3**, the processed gene expression matrix and cell annotation table were obtained from the publication of Liu et al(27). These include scRNA-seq data for the human heart, intestine, pancreatic tumor, and Squamous cell carcinoma. For the colorectal tumor scRNA-seq data used for **Figure 3E-F**, **Supplementary Figure 2A-C**, and **Supplementary Figure 3F-G**, normalized gene expression and cell type annotation were downloaded from the TISCH database (http://tisch.comp-genomics.org/) under the accession "CRC_GSE146771_Smartseq2". The HCT15 and NK coculture CRISPR screen data with sample ID 1657 was downloaded from the BioGRID ORCS (https://orcs.thebiogrid.org/Browse). The downloaded CRISPR screen data was analyzed by MAGeCK RRA algorithm(40) and was the gene level statistics, including scores for both sgRNA enrichment and depletion for every gene. This data generated Figure 3D, 3F, Supplementary Figure 2D-E, and Supplementary Figure 3F. For patient survival analysis using TCGA colorectal tumor samples, we downloaded bulk RNA-seq gene expression data, patient survival statistics, and patient clinic information from UCSC XENA under accession "TCGA Colon and Rectal Cancer (COADREAD) (15 datasets)". The gene expression values in log2(x+1) transformed RSEM normalized count were used. Following the same method implemented in the MEBOCOST, we took the product of expression values of the aggerated enzyme genes and sensor genes to indicate the co-presenting potential of a metabolite-sensor pair in a colorectal tumor RNA-seq sample. The product values were then incorporated in a Cox proportional hazard (Cox-PH) model to assess their effect on the patient's overall survival. The model also factored in patient age and tumor stages to adjust the clinic variations. The hazard ratios derived in this model were used to generate figures related to **Figure 3F** and **Supplementary Figure 3G**, which elucidates the influence of metabolite-sensor pairs on patient survival outcomes.

Linking metabolite-sensor pairs of mCCC with KEGG pathways using bulk RNA-seq in BAT

Modeling the downstream effects of a mCCC to comprehend its potential function is challenging when relying solely on scRNA-seq data. However, it is feasible to associate a specific pathway with a metabolite-sensor pair by examining the correlation between pathway activity and the gene expression of metabolite enzymes and sensors. This can be achieved by analyzing a collection of bulk RNA-seq samples. To link potential pathways with mCCC in BAT, we analyzed 629 mouse brown adipose bulk RNA-seq datasets from the ARCHS4 database(41). We

downloaded the gene-level read count matrix and converted it to TPM (transcripts per million) for normalizing gene length and total read counts. Activities of KEGG pathways were calculated using single sample Gene Set Enrichment Analysis (ssGSEA) with the R package GSVA in all samples. For metabolite-sensor pairs, we determined their activity by calculating the product of gene expression levels of metabolite enzymes and their corresponding sensors in all samples. In cases where a metabolite was associated with multiple enzymes, we aggregated the enzyme gene expressions using the same method as in scRNA-seq analysis. We then assessed the pairwise correlation between the activity scores of metabolite-sensor pairs and pathways across all samples. To prevent artificial correlations caused by shared genes in metabolite-sensor pairs and pathway annotations, we excluded metabolite enzyme and sensor genes from KEGG pathways before ssGSEA. The correlations between metabolite-sensor pairs and pathways, specifically those with 50 to 100 genes, were visualized in a heatmap in **Supplementary Figure 8** and **Supplementary Figure 13**.

Evaluation of MEBOCOST stability

We reasoned that the sequencing depth and the total cell number in scRNA-seq data should influence a good algorithm less. Therefore, we compared the prediction results of subsampling datasets with the result of the original dataset in two aspects using the BAT scRNA-seq data from mice housed at cold temperatures for two days (Cold2). First, we compared the total number of significant communication events between subsampling and original datasets. The communications were deemed significant at an FDR of 0.05. Second, the similarity between the prediction result of subsampling datasets and the original dataset was evaluated. Two overlapping percentages were calculated to measure such similarity. One is the number of overlapped communications between subsampled datasets and the original dataset divided by the number of communications in the subsampled data (recaptured over the total detected). The other one is the number of overlapped communications between subsampled datasets and the original dataset divided by the number of communications in the original dataset (recaptured over the original).

To test the effect of sequencing noise on the performance of MEBOCOST, we added *in silico* noise to the scRNA-seq dataset with a series of noise rates following a Gaussian distribution implemented by the Python package NumPy. Then, the noise rate was calculated as the number of random reads divided by the number of scRNA-seq reads in the original dataset. Each noised dataset was generated by introducing random reads into the Cold2 scRNA-seq

data. The detailed procedures mainly include three steps. First, the total number of noise reads was calculated by multiplying the total read count of Cold2 scRNA-seq data by the given noise rate. Second, data points were randomly selected from the cell-by-gene count matrix. Third, the original read count in the selected data point was added by one. Four, the second and third steps were iterated and stopped until all noise was added to reach the noise rate. The newly generated cell-by-gene count matrix of noised datasets was then subjected to normalization and log transformation by the Scanpy package. Next, MEBOCOST was applied on each noised dataset to detect mCCC.

We also assessed the association between number of cells and the mCCC number detected in sender and receiver cell types, separately. As shown in the **Supplementary Figure 16A-B**, the sender or receiver cell numbers were not associated with the mCCC numbers detected by the MEBOCOST algorithm. We performed the similar analysis to assess the effect of cell numbers of sender and receiver cell types on the mCCC scores in observation and background, respectively. As shown in the **Supplementary Figure 16C-F**, the cell numbers were not correlated with mCCC scores in real observation and background (null distribution). These results suggested that MEBOCOST against the effect of cell population size on the mCCC analysis probably because we computed the mCCC score per cell by taking the averaged gene expression among the cell population.

Identification of the most temperature-sensitive communication events

All the cells of BAT scRNA-seq data from TN, RT, Cold2, and Cold7 were pooled and grouped by cell types and conditions when running MEBOCOST. Communications were deemed as significant if FDR was less than 0.05. The index of dispersion (IOD) was calculated for communication score across the four conditions. Given a sender-metabolite-sensor-receiver combination, the IOD was defined by:

$$IOD = \sigma^2 / \mu$$

where σ^2 is the variance of the four communication scores under the four conditions, and μ is the mean of the four scores. Sender-metabolite-sensor-receiver communications included in this cold-sensitive communication analysis were at least significant in one of the four conditions. The IOD scores of communications were ranked from high to low, and the 100 top-ranked were defined as cold-sensitive communications.

Mice

The C57BL6 mice (strain #:000664) were purchased from the Jackson Laboratory and housed in a temperature-controlled environment (22°C) with a 12-hour light/dark cycle. All experimental procedures involving animals complied with all relevant ethical regulations applied to using small rodents and with approval by the Institutional Animal Care and Use Committees (IACUC) at Joslin Diabetes Center.

Cell Lines

Immortalized mouse brown preadipocytes were generated using SV40 T antigen(42) and cultured in high-glucose DMEM with 10% FBS. Mouse endothelial cells were purchased from ATCC (Cat# C166) and cultured in high-glucose DMEM with 10% FBS.

Primary Stromal Vascular Fraction (SVF) isolation

We obtained the primary adipocyte progenitor cells from SVF of interscapular BAT of 8-week-old male C57BL6 mice as previously described(35). BAT was dissected, minced with scissors, and digested in digestion buffer containing type 1 collagenase (1.5 mg/ml; Worthington Biochemical), dispase II (2.5 U/ml; Stemcell Technologies), fatty acid-free BSA (2%; Gemini Bio-Products) in Hanks' balanced salt solution (Corning Hanks' Balanced Salt Solution, 1× with calcium and magnesium) for 45 minutes at 37 °C with gentle shaking. The digested samples were filtered through a 200-micron gauge mesh and centrifuged at 500 rpm for 5 minutes at room temperature. SVF pellets were resuspended in RBC lysis buffer (Sigma, Cat# R7757) to remove red blood cells by incubating the cell suspension for 5 minutes at room temperature. PBS was added to quench the reaction, and the SVF pellet was collected by centrifugation at 500 rpm for 5 minutes at room temperature. The SVF pellet was resuspended in an appropriate volume of growth medium (High glucose DMEM with 10% FBS) and seeded onto plates.

Cell proliferation assay

A total of 0.5 x 10^3 primary SVF cells were seeded into wells of 96-well white-wall plates and cultured for four days in a glutamine-free growth DMEM medium containing 10% dialyzed FBS (ThermoFisher Scientific, 26400044) supplemented with vehicle or 300 μM L-glutamine, or a combination of 300 μM L-glutamine and 10 μM V9302. At indicated time points, cells were incubated with an equal volume of CellTiter-Glo 2.0 Reagent (Promega, Cat# G9242) for 10 minutes at room temperature. The luminescence signal was recorded with GloMax Discover Microplate Reader (Progema) using the CellTiter-Glo protocol. The cell proliferation rate was calculated by normalizing the luminescence signal to the Day 0 signal.

Preadipocytes differentiation

To induce differentiation, immortalized brown preadipocytes were allowed to reach confluency and were treated with an induction medium supplemented with 10% FBS, 20 nM insulin, 1 nM Triiodothyronine (T3), 0.125 mM indomethacin, 5 μM dexamethasone, and 0.5 mM IBMX for 2 days. Subsequently, cells were maintained in a differentiation medium supplemented with 2% FBS, 20 nM insulin, 1 nM T3 for an additional 4 days. During differentiation, the medium was changed every other day. The base medium for induction and differentiation medium was prepared using glutamine-free DMEM supplemented with 1 mM MSO (Methionine sulfoximine). To assess the effect of glutamine on differentiation, the cells were treated with vehicle, 300 μM L-glutamine, or a combination of 300 μM L-glutamine and 10 μM V9302..

Oil Red O staining

Adipocytes were washed twice with PBS and fixed with 10% formalin for 30 minutes at room temperature. Cells were then rinsed twice with distilled water (dH2O) and incubated with 60% Isopropanol for 5 minutes at room temperature. For lipid staining, cells were entirely and evenly covered with Oil Red O working solution (a mixture of 3 parts 0.5% Oil Red O in isopropanol and 2 parts dH2O) for 1 hour at room temperature, followed by five washes with dH2O. The stained cells were then visualized. To quantify lipid accumulation level, Oil Red O was solubilized with 100% isopropanol, and the absorbance values at 492 nm were measured using GloMax Discover Microplate Reader (Progema).

Endothelial cell-released glutamine measurement

Mouse endothelial cells (ATCC, Cat# C166) were cultured in glutamine-free DMEM supplemented with 10% dialyzed FBS (ThermoFisher Scientific, 26400044) for 24 hours. The supernatant was collected, and insoluble particles were removed by centrifugation at 10,000 g for 10 minutes at 4°C. The supernatant was further filtered with a 10 kDa spin filter to deproteinate. The flow-through was collected and utilized for the downstream glutamine measurement, which was carried out using a Glutamine Assay Kit (CELL BIOLABS, INC. Cat# MET-5165). The fluorescence was recorded using GloMax Discover Microplate Reader (Progema) with excitation at 520 nM and emission at 590 nM. The relative fluorescence units were converted to glutamine concentrations in μM based on the standard curve following the instructions provided by the kit, the resulted negative values were replaced by zero as

concentrations should be non-negative values. Glutamine-free DMEM supplemented with 10% dialyzed FBS was used as background for the measurement.

RNA extraction and qRT-PCR

Total RNA from mature adipocytes was extracted using Direct-zol RNA Miniprep kits (Zymo Research, R2050). cDNA synthesis was carried out using the High-Capacity RNA-to-cDNA™ kit (Applied Biosystems™, ThermoFisher Scientific, 4368814) with random hexamers. qRT-PCR was performed using an ABI Prism 7900 sequence-detection system with. The relative expression of genes was normalized to the housekeeping gene ARBP mRNA. Primers (ARBP F: TTTGGGCATCACCACGAAAA, ARBP R: GGACACCCTCCAGAAAGCGA; Fabp4 F: GATGCCTTTGTGGGAACCT, Fabp4 R: CTGTCGTCTGCGGTGATTT; Pparg F: TCAGCTCTGTGGACCTCTCC, Pparg R: ACCCTTGCATCCTTCACAAG; Prdm16 F: CAGCACGGTGAAGCCATTC, Prdm16 R: GCGTGCATCCGCTTGTG) used in qRT-PCR were synthesized from IDT.

Overexpression of *Glul* in endothelial

To construct the *Glul* overexpression plasmid, the full-length *Glul* cDNA was amplified and cloned into the pLVX vector (Takara Bio, PT4002-5) using XhoI and ApaI. Lentiviral particles were produced in HEK293T cells by co-transfection of *Glul* construct with packaging plasmids, psPAX2 (Addgene, plasmid# 12260, a gift from Dr. Didier Trono) and pMD2.G (Addgene, plasmid# 12259, a gift from Dr. Didier Trono). Following 24 hours of transfection, the medium was replaced with prewarmed DMEM medium. After an additional 48 hours, culture media containing lentiviral particles were collected, filtered through a 0.45 μM filter, aliquoted, and stored at -80°C. To overexpress *Glul*, endothelial cells were infected with lentivirus expressing *Glul* and selected with Geneticin selective antibiotic for 7 days. The overexpression efficiency was validated by western blotting, and empty vector was used as a control.

Knockdown of SLC1A5 and SLC38A2 in preadipocytes with CRISPR/Cas9

Two gRNAs targeting Slc1a5 (GGTCACCACACTGCTCGCGT, CCGCAGTGCACCAACCAAAG), two gRNAs targeting Slc38a2 (CTGCATGGTTATCGATCCGG, AGTCTTGAGGAGGAGATGGA), and a non-targeting control gRNA (TCTGATAGCGTAGGAGTGAT) were individually inserted into lentiCRISPRv2 neo (Addgene, plasmid# 98292, a gift from Dr. Brett Stringer) and packaged into lentiviral particles. Murine brown preadipocytes were transduced with lentivirus expressing individual gRNA and

selected with Geneticin selective antibiotic. The knockdown efficiency of each gRNA was validated by western blotting.

Western blotting

Transduced endothelial cells were lysed in RIPA buffer (Boston BioPruducts Inc., BP-115) supplemented with 1x protease inhibitor cocktail (Millipore Sigma, P8340). Protein concentration was determined by BCA analysis. Protein concentration was determined by BCA analysis (ThermoFisher Scientific, 23225). Protein lysates were denatured in 4x Laemmli sample buffer (Bio-Red, 1610747), resolved by 4%-20% Mini-PROTEAN TGX^{TM} precast protein gels, and transferred to a 0.22 μM pore size nitrocellulose (NC) membrane. Primary antibody (GLUL: Cell signaling, 80636; SLC1A5: Proteintech, 2035-1-AP; SLC38A2: Proteintech, 25928-1-AP) was diluted in TBST containing 1% BSA. Membranes were incubated with each primary antibody overnight at 4°C. Anti-Rabbit secondary antibody (Cell Signaling, 7074) was diluted in TBST containing 1% milk. Membranes were visualized with SuperSingal^{TM} West Femto Maximum Sensitivity Substrate (ThermoFisher Scientific, 34094) using Bio-Red ChemiDoc Imaging System.

Metabolomics Analysis

Plasma and tissue samples were collected from mice with thermoneutrality (30 °C) or cold exposure (5 °C) for 4 hours and used for metabolomics analysis performed as previously described(43). Metabolite extraction was achieved using a mixture of isopropanol, acetonitrile, and water at a ratio of 3:3:2 v/v. Extracts were divided into three parts: 75 uL for gas chromatography combined with time-of-flight high-resolution mass spectrometry, 150 uL for reversed-phase liquid chromatography coupled with high-resolution mass spectrometry, and 150 uL for hydrophilic interaction chromatography with liquid chromatography and tandem mass spectrometry, and analyzed as previously described. We used the NEXERA XR UPLC system (Shimadzu, Columbia, MD, USA), coupled with the Triple Quad 5500 System (AB Sciex, Framingham, MA, USA) to perform hydrophilic interaction liquid chromatography analysis, NEXERA XR UPLC system (Shimadzu, Columbia, MD, USA), coupled with the Triple TOF 6500 System (AB Sciex, Framingham, MA, USA) to perform reversed-phase liquid chromatography analysis, and Agilent 7890B gas chromatograph (Agilent, Palo Alto, CA, USA) interfaced to a Time-of-Flight Pegasus HT Mass Spectrometer (Leco, St. Joseph, MI, USA). The GC system was fitted with a Gerstel temperature-programmed injector, a cooled injection system (model CIS 4). An automated liner exchange (ALEX) (Gerstel, Muhlheim an der Ruhr, Germany) was

used to eliminate cross-contamination from the sample matrix that was occurring between sample runs. Quality control was performed using a metabolite standards mixture and pooled samples applying the methodology previously described(44). A quality control sample containing a standard mixture of amino and organic acids purchased from Sigma-Aldrich as certified reference material, was injected daily to perform an analytical system suitability test, and monitor recorded signals day to day reproducibility as it was described. A pooled quality control sample was obtained by taking an aliquot of the same volume of all samples from the study and injecting daily with a batch of analyzed samples to determine the optimal dilution of the batch samples and validate metabolite identification and peak integration. Collected raw data were manually inspected, merged, imputed, and normalized by the sample median. Metabolite identification was performed using in house authentic standards analysis. Metabolite annotation was used utilizing recorded retention time and retention indexes, recorded MSn and HRAMSn data matching with METLIN, NIST MS, Wiley Registry of Mass Spectral Data, HMDB, MassBank of North America, MassBank Europe, Golm Metabolome Database, SCIEX Accurate Mass Metabolite Spectral Library, MzCloud, and IDEOM databases.

Supplementary Figures:

Supplementary Figure 1

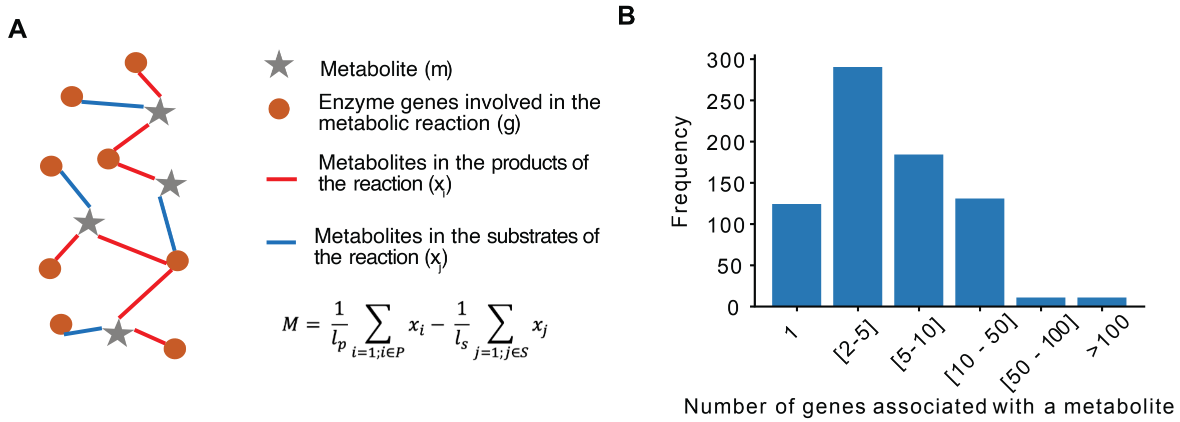

Supplementary Figure 1. Aggregation of metabolite enzymes based on RNA expression. **A**. The schematic plot showed the method to aggregate the RNA expression values of metabolite enzymes. The network showed that each metabolite (star) could be produced or consumed by multiple enzymes (dot) in the generation reactions (red lines) and consumption reactions (blue lines), respectively. **B**. Number of metabolites plotted against the number of associated enzymes per metabolite.

Supplementary Figure 2

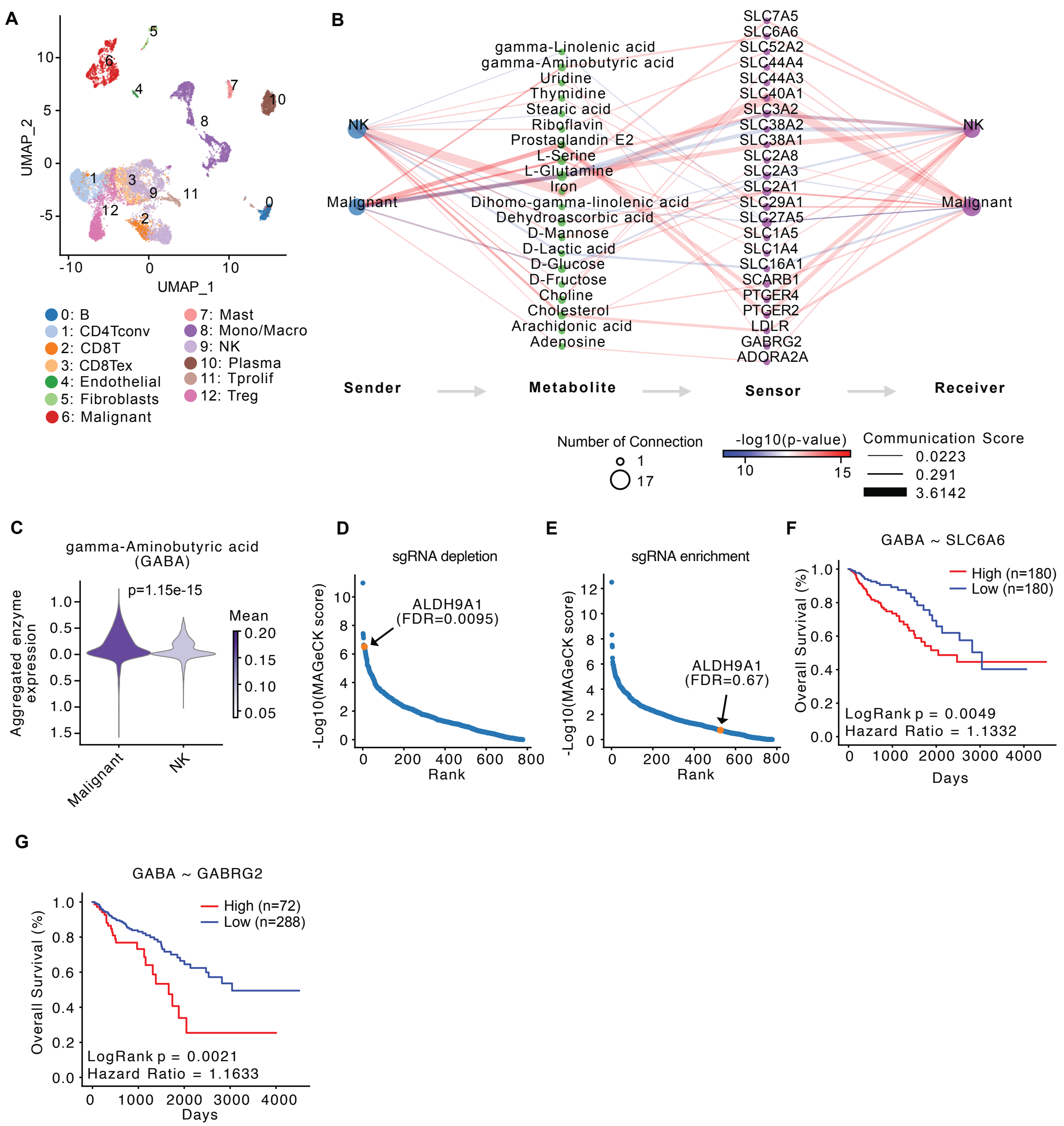

Supplementary Figure 2. Using CRISPR screen and patient survival data as orthogonal information to compare MEBOCOST and other general CCC methods.

A. A UMAP plot to show the cellular composition in the colorectal tumor scRNA-seq sample (GSE146771). **B**. A flow diagram showing mCCC from sender to receiver cell types identified between malignant and NK cells in colorectal tumors. The dot size represents the number of connections in the diagram. The lines connect sender cells, metabolites, sensors, and receiver

cells. Line width represented the mCCC score. The line color indicates the -log10(FDR) of a communication event. **C**. Violin plot shows the GABA enzyme genes were highly expressed in sender cells (malignant cells) than receiver cells (NK cells). **D-E**. GABA enzyme ALDH9A1 was a top ranked enzyme with significant sgRNA depletion (**D**) and non-significant sgRNA enrichment (**E**) in cancer-NK coculture CRISPR screen result. **F-G**. Patient survival analysis was performed using the product of GABA enzyme gene expression and sensor gene expression in TCGA colon cancer cohort. **F** shows results for sensor SLC6A6, and **G** shows another sensor GABRG2. Patient groups were separated based on median values.

Supplementary Figure 3

A

Method	# of metabolite-sensor pairs	Neurotransmitter	Extracellular metabolites beside neurotransmitter	Sensors for metabolites			Efflux / influx
				Receptor	Transporter	Nuclear receptor	
MEBOCOST	440	√	√	√	√	√	√
CellPhoneDB	194	√	√	√	×	√	×
scConnect	50	√	×	√	×	×	×
NeuronChat	221	√	×	√	√	×	×

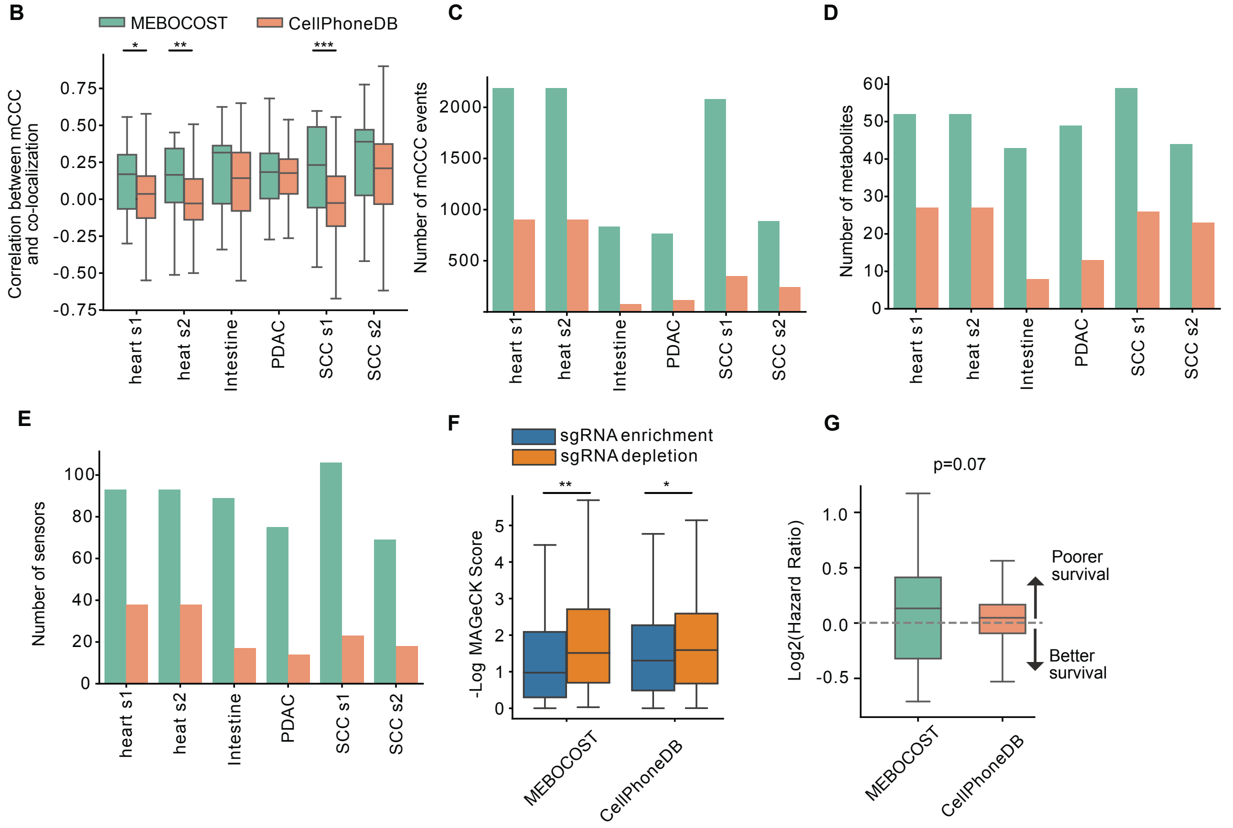

Supplementary Figure 3. Using spatial transcriptomics data as orthogonal information to compare MEBOCOST and other general CCC methods.

A. A table summarizing key features of MEBOCOST, CellPhoneDB, scConnect, and NeuronChat. **B.** A box plot showing the correlation between cell-cell communication scores and cell-cell colocalization scores. Metabolites that each mediated communications between 10 or more different pairs of cell types were analyzed. P value determined by one-tailed t-test. * denotes p-value < 0.05, ** denotes p-value < 0.01. **C-E.** Bar plots to show the number of detected mCCC events in different samples (**C**) and the number of metabolites (**D**) and sensors (**E**) in the detected mCCC events. **F.** A boxplot to show the scores for sgRNA depletion (orange

box) and enrichment (blue box) for genes (enzymes and sensors) in mCCC events detected by MEBOCOST and CellPhoneDB. The Y axis shows the minus log-transformed scores for sgRNA depletion or enrichment computed by the MAGeCK software. A one-tailed Wilcoxon signed-rank test was used to test the statistical difference (p-value) between sgRNA enrichment and sgRNA depletion. * denotes p-value < 0.05, ** denotes p-value < 0.01. **G**. A box plot showing the log2 hazard ratio of TCGA patients computed using expression of enzyme and sensor genes between those from significant and non-significant mCCC events detected by MEBOCOST and CellPhoneDB. A positive log2 hazard ratio suggests that the gene expression is associated with worse patient survival, while a negative log2 hazard ratio suggests that the gene expression is associated with better survival. A one-tailed T-test was used to compute the p-value between the log2 hazard ratio of significant mCCC and non-significant mCCC.

Supplementary Figure 4

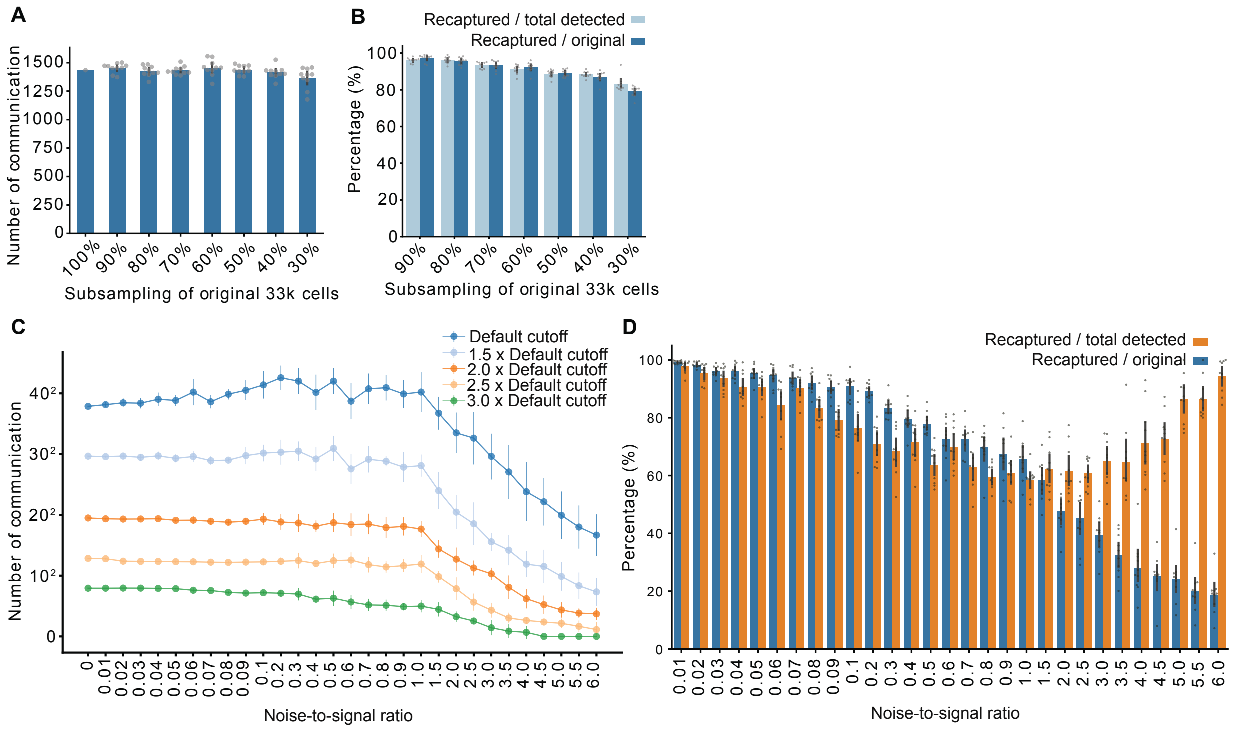

Supplementary Figure 4. Simulation analysis demonstrated performance stability and great scalability of MEBOCOST.

A-**B**. Bar plots to show the number of detected communications (**A**) and recapture rate (**B**) of MEBOCOST when applied to a series of down-sampled scRNA-Seq datasets. The error bar showed a range of results from 10 repeats of down-sampling experiments. **C** and **D.** The number of detected communications (**C**) and recapture rate (**D**) plotted against simulated noise-to-signal ratio after applying MEBOCOST to a series of simulated scRNA-seq data. The original sequenced reads were recognized as the signal, whereas noise was simulated by adding random reads to the scRNA-seq data. The error bar showed the range of results from 10 repeats of the simulation experiments. All down sampling and simulation experiments were performed based on mice BAT data from the Cold2 condition.

Supplementary Figure 5

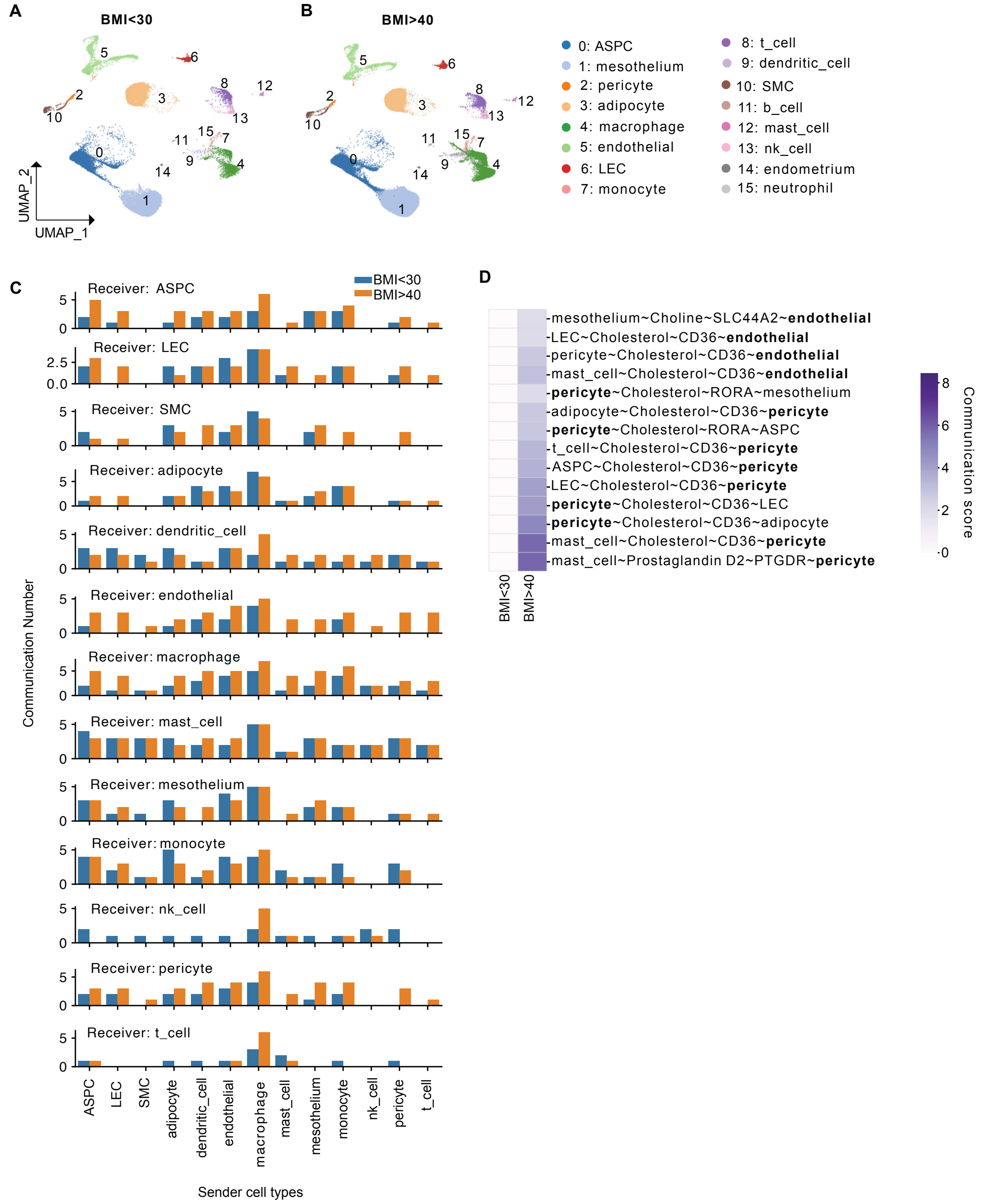

Supplementary Figure 5. Human WAT scRNA-seq data and mCCC dynamics during obesity.

A-B. UMAP plots of scRNA-seq data of WAT from individuals with BMI < 30 and BMI > 40. **C.** The number of mCCC detected by MEBOCOST. The X-axis represents the sender cell types. Each row represented a receiver cell type. The Y-axis showed the number of mCCC between sender and receiver cell types. **D.** A heatmap to show the increased endothelial-related or pericyte-related mCCC (difference of communication scores between BMI>40 and BMI<30 samples greater than 2). The color represented the communication score. Each row showed a mCCC event.

Supplementary Figure 6

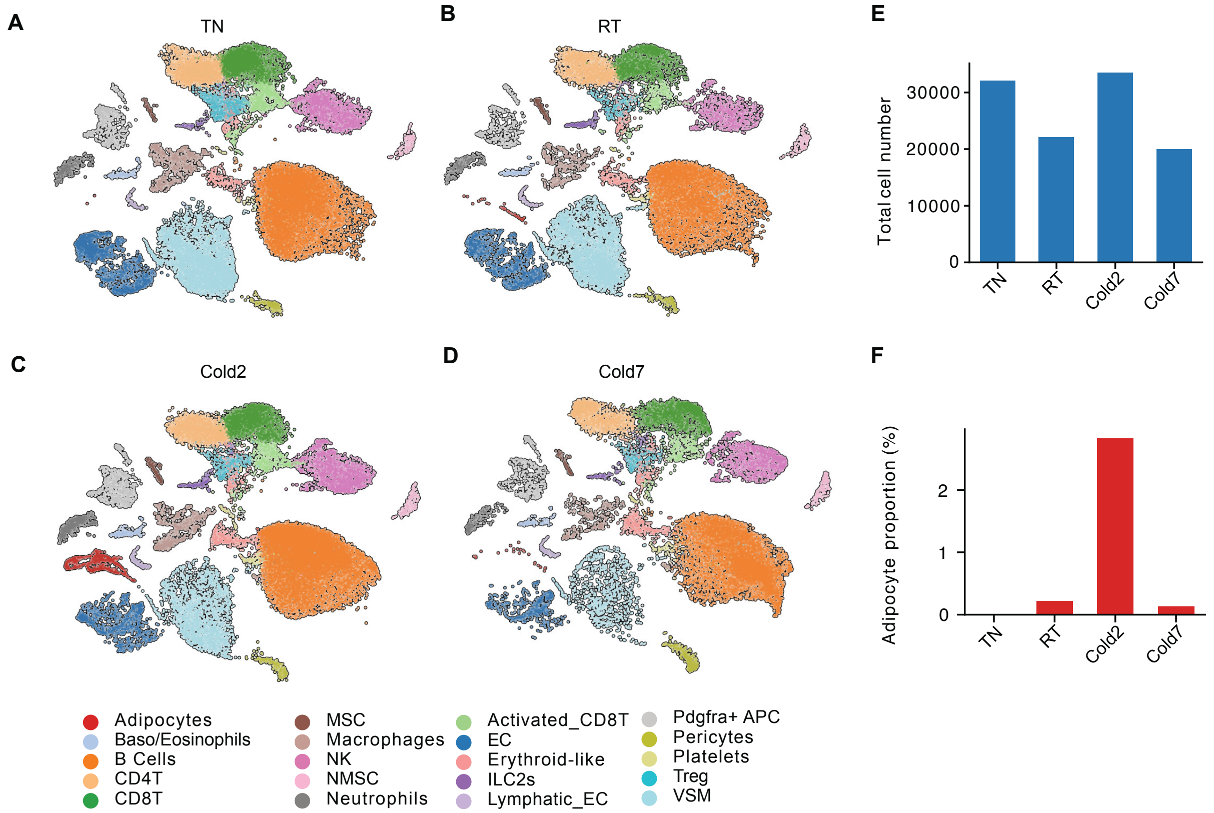

Supplementary Figure 6. Single-cell RNA-seq analysis of mice brown adipose tissues. A-D. UMAP visualization of the scRNA-seq data of BAT from four conditions, including TN (30 °C for a week) (A), RT (room temperature) (B), Cold2(5 °C for 2 days) (C), and Cold7 (5 °C for 7 days) (D). **E**. The total number of cells in the scRNA-seq data from the four conditions. **F**. The proportion of differentiating adipocytes across the four conditions.

Supplementary Figure 7

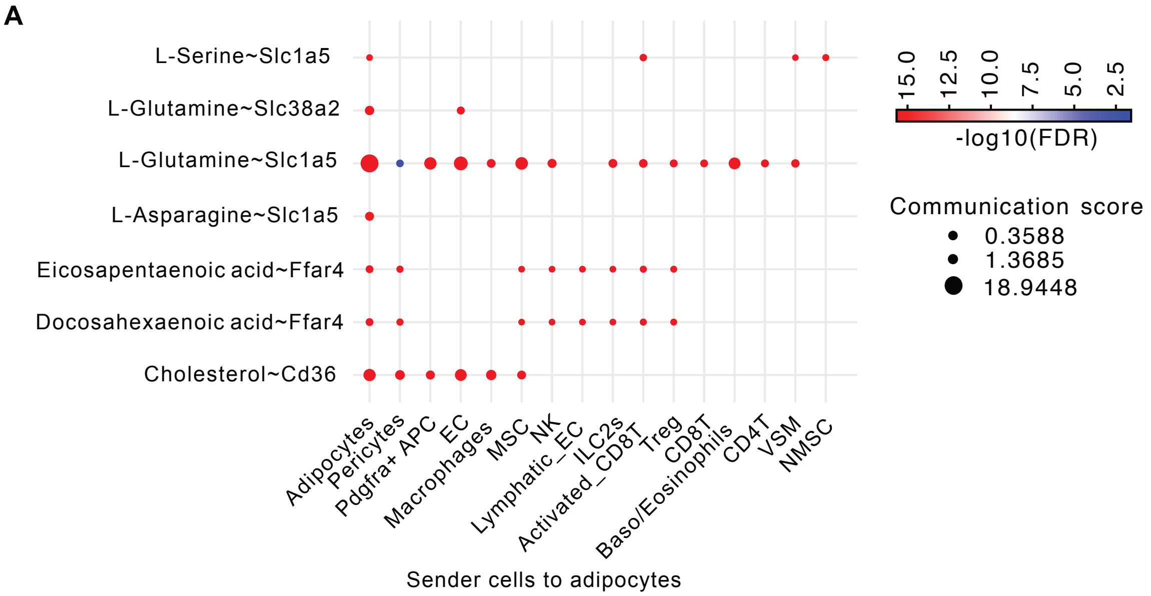

Supplementary Figure 7: Metabolite-sensor communications sending to brown adipocytes in Cold2 BAT

A. A dot plot showing metabolite-sensor communications send to adipocytes from adipocytes themselves and other cell types. The rows represent metabolite-sensor partners; the columns represent the sender cell types in the communications with adipocytes. The dot size indicates the communication score calculated by MEBOCOST. The color of the dot was the -log10(FDR) for the metabolite-sensor communication score.

Supplementary Figure 8

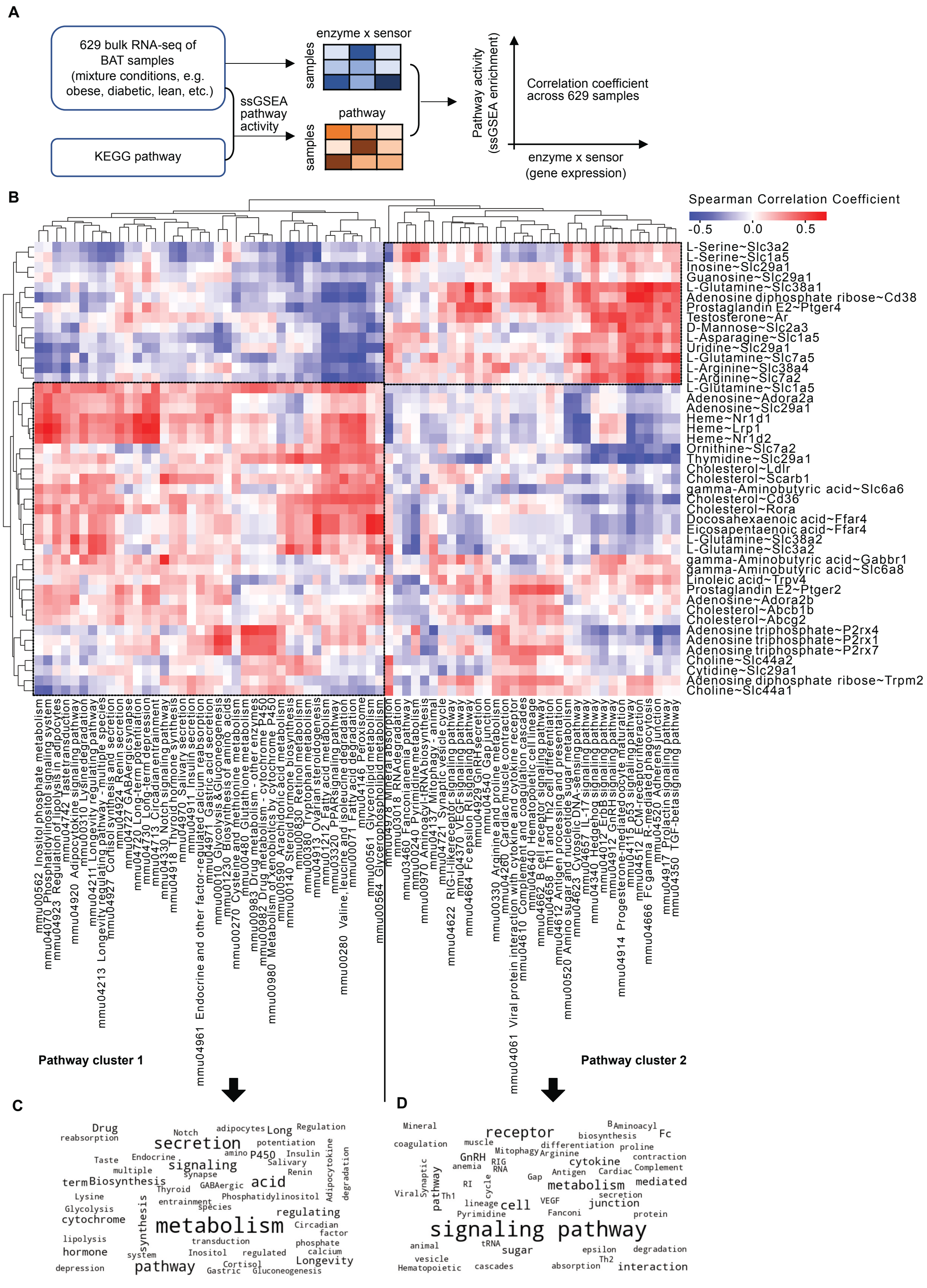

Supplementary Figure 8. Integrating bulk RNA-seq to associate KEGG pathways with mCCC in Cold2 BAT.

A. A schematic of association analysis between mCCC and pathways using bulk RNA-seq of BAT samples. **B.** The heatmap shows the associations between KEGG pathways and metabolite-sensor pairs in significant mCCC of the Cold2 BAT sample. The value represents the Spearman correlation coefficient between pathway enrichment scores and metabolite enzyme-sensor expression across 629 BAT RNA-seq samples. Rows are metabolite-sensor pairs, and columns are KEGG pathways. Clustering was performed by rows and columns based on Euclidean distance. The two clusters (pathway cluster 1 and pathway cluster 2) labeled by a dashed black box were determined based on the clustering result for columns. **C**-**D**. Word-cloud plots of the KEGG pathway terms corresponding to the two clusters in B.

Supplementary Figure 9

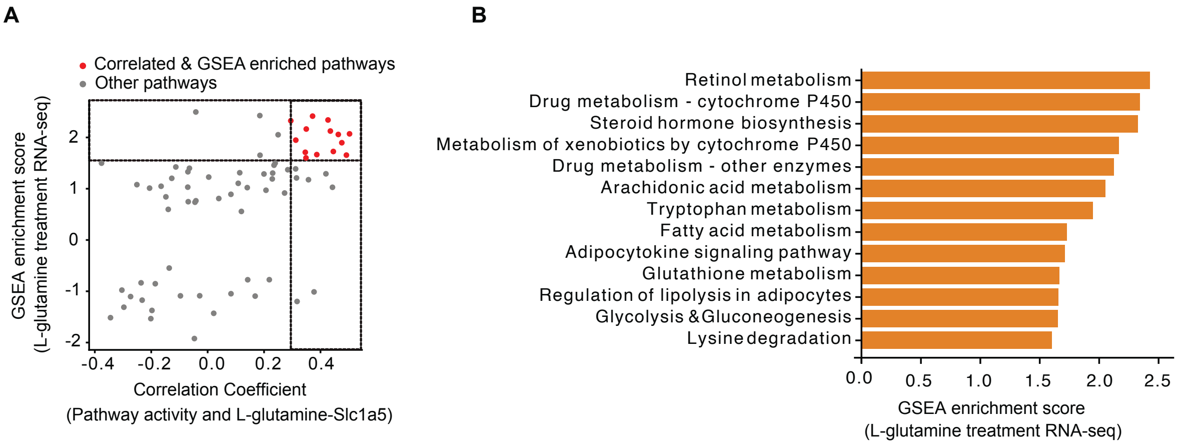

Supplementary Figure 9. Up-regulated pathways upon L-glutamine treatment in brown adipocytes

A. Comparison between correlation analysis and GSEA analysis. The x-axis represents correlation coefficient between pathway activity and L-glutamine-Slc1a5 expression. The y-axis represented the pathway enrichment score in brown adipocyte RNA-seq dataset with L-glutamine treatment. **B**. The bar plot shows pathway enrichment score for those with high correlation coefficient and GSEA enrichment score in panel A.

Supplementary Figure 10

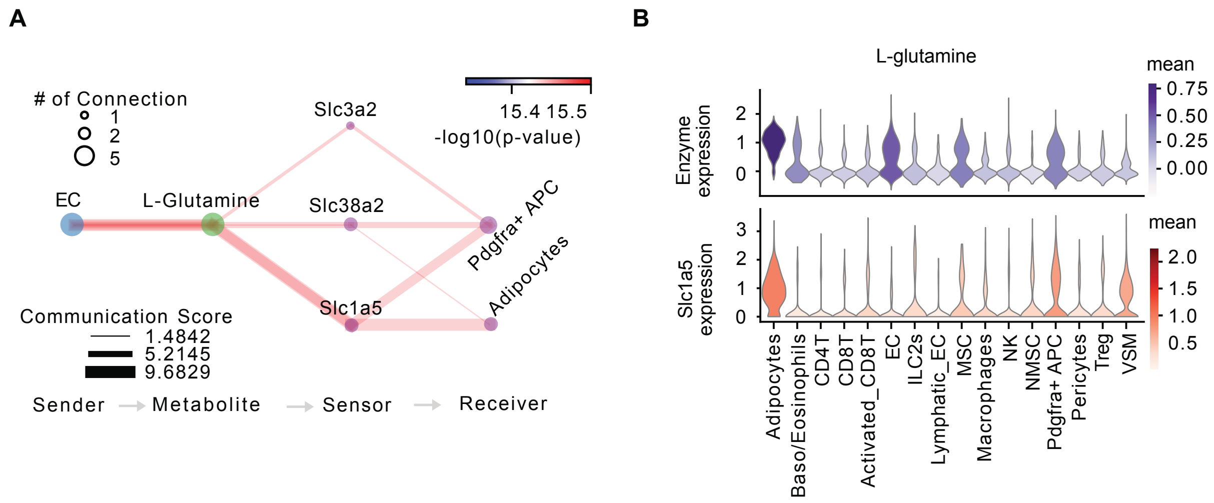

Supplementary Figure 10. Glutamine mediates communications to brown adipocytes. **A.** A flow diagram showing mCCC from sender cell types to receiver cell types identified between EC and adipocytes in Cold2 BAT samples. The dot size represents the number of connections in the diagram. The lines connect the sender, metabolites, sensors, and receiver cells. The line width represented the metabolite-sensor communication score. The line color indicated the -log10(FDR) of a communication event. **B.** Violin plots showing the RNA expression of enzymes (top panel) and sensor proteins (bottom panel) of metabolites in individual cell types of brown adipose tissue.

Supplementary Figure 11

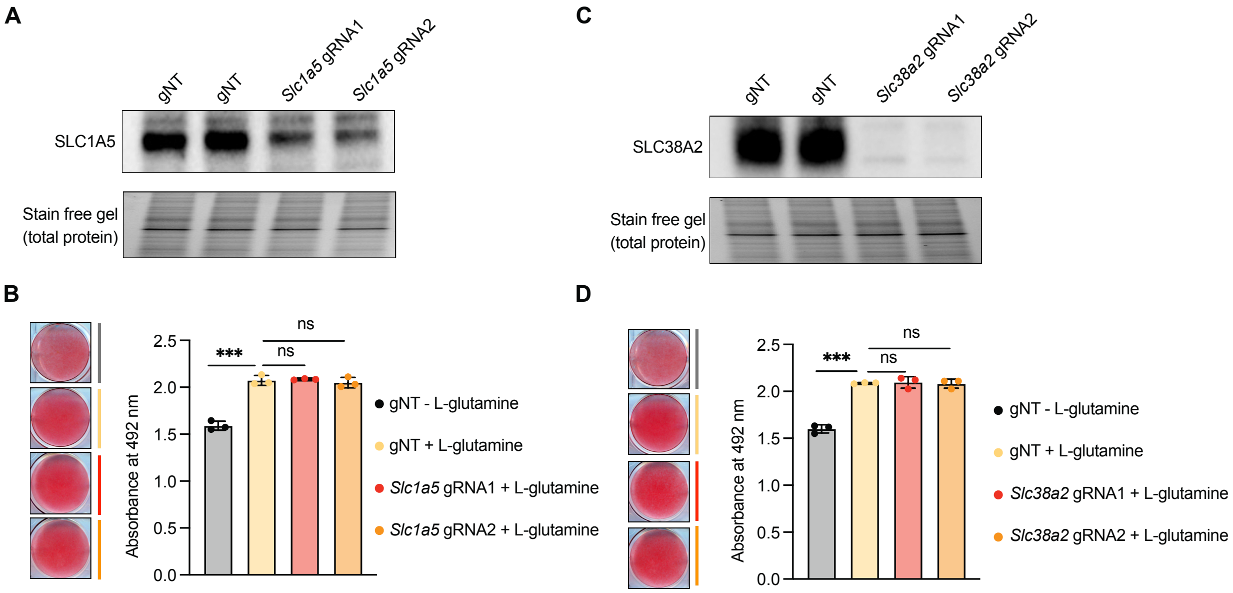

Supplementary Figure 11. Knockdown of SLC1A5 and SLC38A2 to verify glutamine sensors in brown adipocytes via CRISPR/Cas9.

A. The western-blot analysis showing the SLC1A5 protein level in preadipocytes with non-targeted guide RNA (gNT) and two *Slc1a5* specific targeted guide RNAs (gRNA1 and gRNA2). Total proteins from the stain-free gel were used as loading control. **B**. The western-blot analysis showing the SLC38A2 protein level in preadipocytes with non-targeted guide RNA (gNT) and two *Slc38a2* specific targeted guide RNAs (gRNA1 and gRNA2). Total proteins from the stain-free gel were used as loading control. **C-D**. The Oil-Red O staining for the lipid accumulation for the adipocyte differentiation analysis with vehicle or L-glutamine treatment in non-targeted gNT and gene targeted gRNA groups. The **C** and **D** showed the result for SLC1A5 and SLC38A2 knockdown experiments, respectively. The left panel in **C** and **D** showed the representative images of Oil-Red O staining, and right panel showed the quantification of Oil-Red O staining that measured the absorbance at 492 nm. *** denotes p-value < 0.001 of two-tailed t-test. ns: non-significant.

Supplementary Figure 12

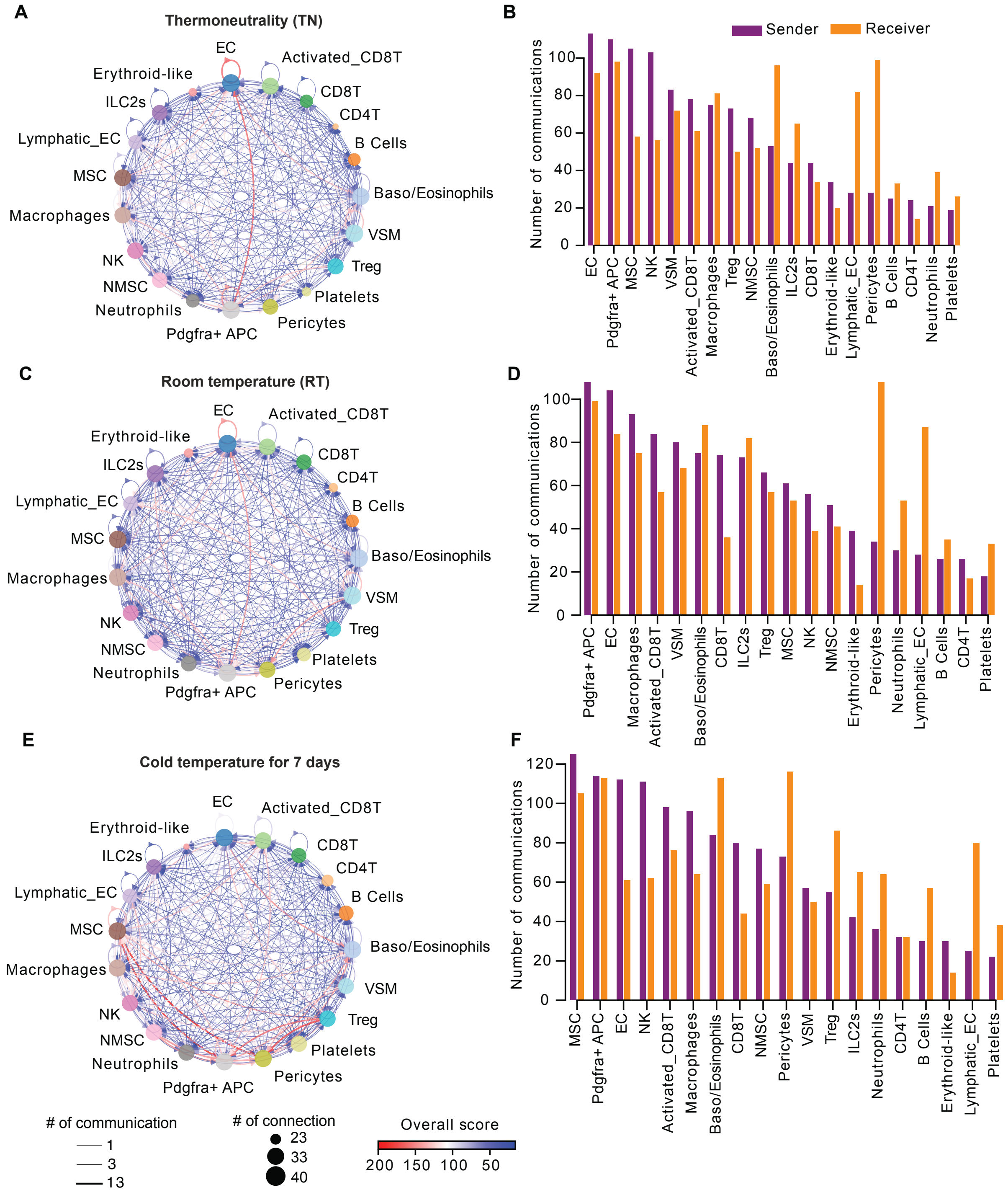

Supplementary Figure 12. The mCCC events detected by MEBOCOST in BAT of mice from the TN, RT, and Cold7 conditions.

A, C, E. Network plot showing mCCC detected by MEBOCOST for mouse BAT at the TN (**A**), RT (**C**), and Cold7 (**E**) conditions. Each dot was a cell type. The dot size for each cell type represented the number of communications with the other cell types. Each arrow line represented the communication from a sender cell type to a receiver cell type. The line width indicated the number of metabolite-sensor communications between the sender and receiver cell types. The line color showed the overall communication score calculated by the sum of -log10(FDR) of all metabolite-sensor communications between the sender and receiver types. **B, D, F**. Bar plot to show the number of detected communications with each cell type as sender cells or receiver cells at the TN (B), RT (D), and Cold7 (F) conditions.

Supplementary Figure 13

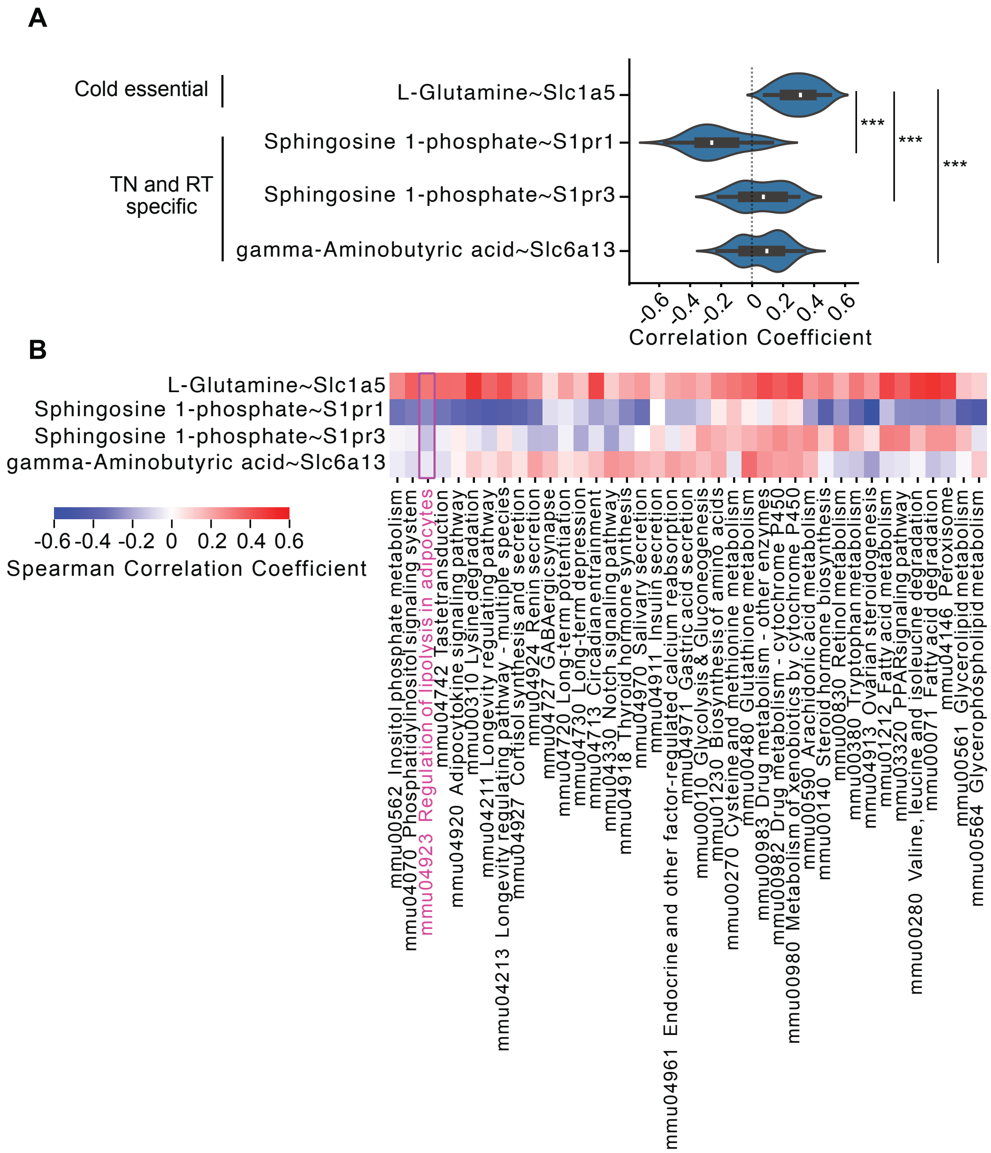

Supplementary Figure 13. TN and RT specific metabolite-sensor partners showed different pathway associations from Cold2 essential metabolite-sensor.

A. Violin plots show overall correlation coefficients between pathway and metabolite-sensor partners for previously identified pathway cluster 1. Three TN and RT specific metabolite-sensor partners were analyzed along with a Cold2 essential metabolite-sensor partner. **B**. Heatmap shows the associations between KEGG pathways and individual metabolite-sensor pairs.

Supplementary Figure 14

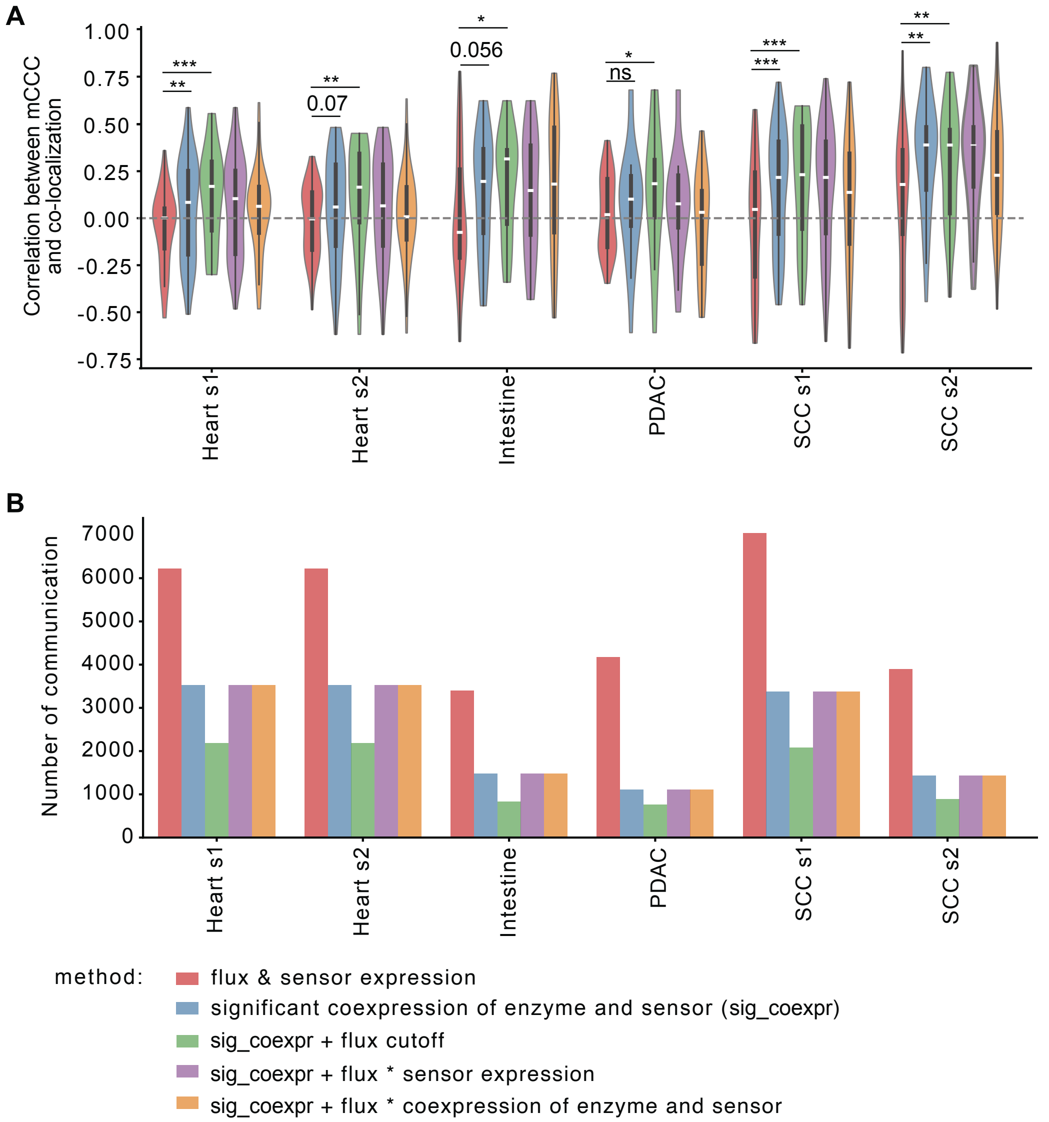

Supplementary Figure 14. Using spatial transcriptomics data to evaluate the different options for mCCC analysis.

A. A violin plot showing the correlation between mCCC scores and cell-cell colocalization scores in different samples. P values was labeled and were determined by a one-tailed t-test. *, p-value < 0.05, **, p-value < 0.01, ns, non-significant. **B**. A bar plot showing the number of mCCC in different samples. The color in both plots showed different methods for mCCC analysis. “flux & sensor expression” is the method for detecting mCCC based on efflux/influx rates and sensor gene expression. “significant co-expression of enzyme and sensor” means the method based on significant co-expression of metabolite enzymes and sensors, also refers to “sig_coexpr”

method. “sig_coexpr + flux cutoff” means the method based on efflux/influx rates and significant enzyme-sensor co-expression. “sig_coexpr + flux * sensor expression” means the method based on efflux/influx rates, sensor gene expression, and significant enzyme-sensor co-expression. “sig_coexpr + flux * co-expression of enzyme and sensor” means the method based on efflux/influx rates, enzyme-sensor co-expression, and the expression of both sensors and metabolic enzymes.

Supplementary Figure 15

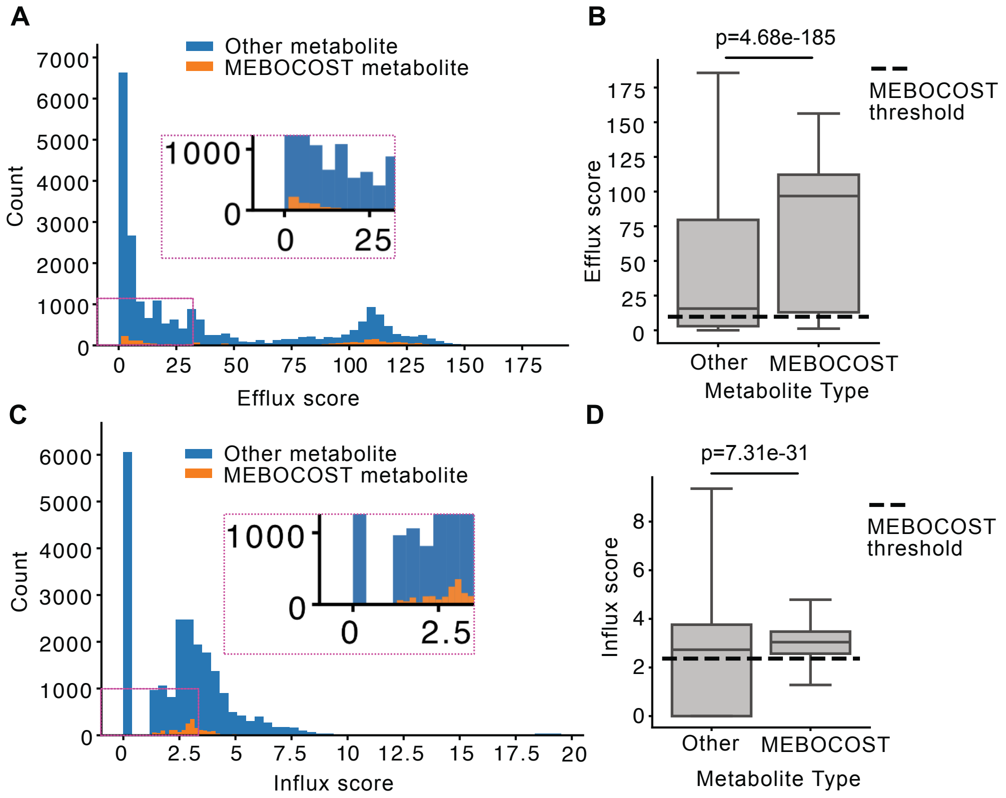

Supplementary Figure 15. The comparison of efflux or influx scores of MEBOCOST metabolites with other metabolites.

A and **C** showed the histograms for efflux (A) and influx scores (B), respectively, computed using COMPASS on brown adipose tissue scRNA-seq data. The purple boxes showed the zoom in on the values around zero. **B** and **D** showed the box plots for efflux (B) and influx (D) comparison between metabolites in the MEBOCOST database and other metabolites. The black dashed lines showed the default flux threshold used in MEBOCOST.

Supplementary Figure 16

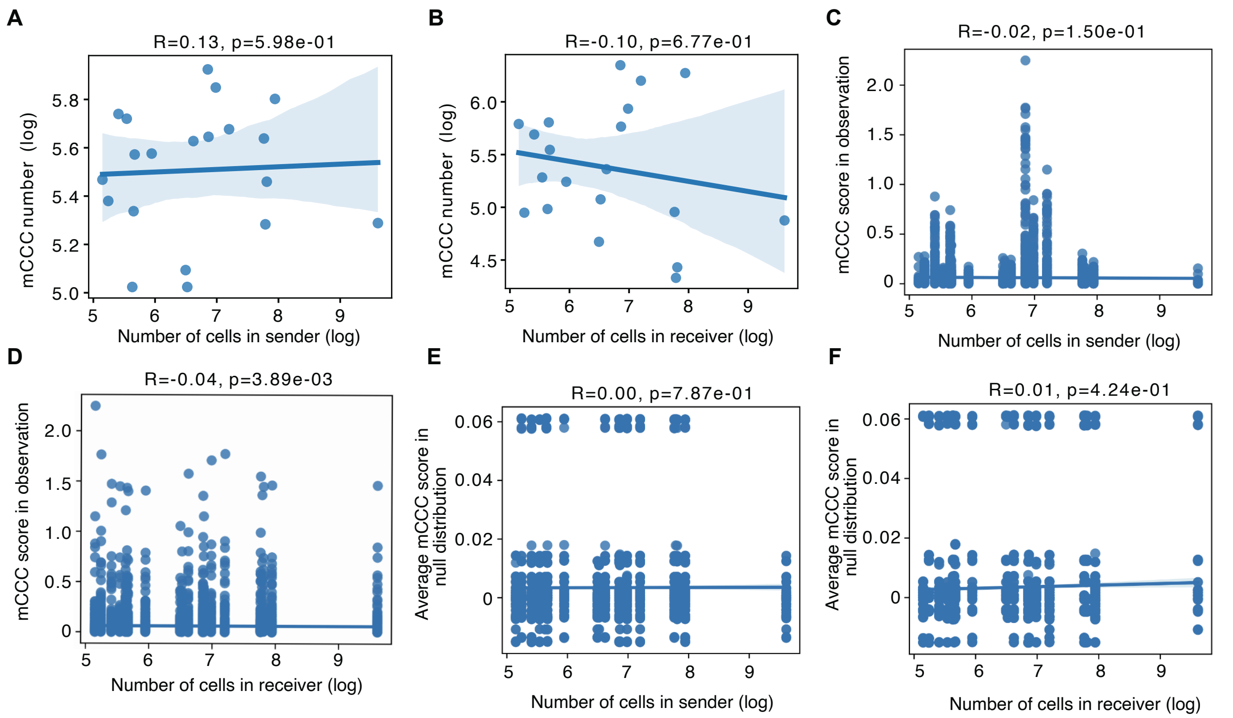

Supplementary Figure 16. The effects of cell population size on mCCC analysis. Scatter plots to show the effects of cell numbers in cell types on communication numbers (**A** for senders and **B** for receivers), communication scores in observation (**C** for senders and **D** for receivers), and communication scores in background distribution (**E** for senders and **F** for receivers). The correlation coefficient and p values were shown on the top of each plot.

Supplementary Tables

Supplementary Table 1. Metabolite-sensor partners collected in MEBOCOST database.

The table contains metabolite-sensor pair with evidence. The Evidence column include PMID, database name, or source links for the collection. The “Evidence_Score” was computed by number of source or PMID. “Text_Evidence” column shows the sentence in the abstract of a publication for judging the metabolite-sensor relationship. N/A means the pair may be collected from a database, website, a publication table, or figures.

Supplementary Table 2. The detected mCCC events in BAT scRNA-seq samples.

The table contains four sheets representing the detected mCCC events in the TN (thermoneutrality), RT (room temperature), Cold2 (cold exposure for 2 days), and Cold7 (cold exposure for 7 days). Each sheet includes columns for sender cell types, metabolite accession numbers in the HMDB database, metabolite names, receiver cell types, sensor names, communication scores, and FDR values computed based on *p*-values of the permutation test.

Supplementary Table 3. A summary of public datasets used in this study.

The table contains public repositories and data sources used in this study. The public repositories include those where we collected extracellular metabolites, metabolite enzymes, sensors, and partners of metabolites and sensors. The data sources include those where we downloaded data for analyses, including bulk RNA-seq, scRNA-seq, spatial transcriptomics, CRISPR screen, and cancer patient survival data.

Supplementary References

1. Browaeys, R., Saelens, W. and Saeys, Y. (2020) NicheNet: modeling intercellular communication by linking ligands to target genes. *Nat Methods*, **17**, 159-162.
2. UniProt, C. (2021) UniProt: the universal protein knowledgebase in 2021. *Nucleic Acids Res*, **49**, D480-D489.
3. Saier, M.H., Reddy, V.S., Moreno-Hagelsieb, G., Hendargo, K.J., Zhang, Y., Iddamsetty, V., Lam, K.J.K., Tian, N., Russum, S., Wang, J. *et al.* (2021) The Transporter Classification Database (TCDB): 2021 update. *Nucleic Acids Res*, **49**, D461-D467.
4. Saier, M.H., Jr., Reddy, V.S., Tamang, D.G. and Vastermark, A. (2014) The transporter classification database. *Nucleic Acids Res*, **42**, D251-258.
5. Saier, M.H., Jr., Reddy, V.S., Tsu, B.V., Ahmed, M.S., Li, C. and Moreno-Hagelsieb, G. (2016) The Transporter Classification Database (TCDB): recent advances. *Nucleic Acids Res*, **44**, D372-379.
6. Saier, M.H., Jr., Tran, C.V. and Barabote, R.D. (2006) TCDB: the Transporter Classification Database for membrane transport protein analyses and information. *Nucleic Acids Res*, **34**, D181-186.
7. Saier, M.H., Jr., Yen, M.R., Noto, K., Tamang, D.G. and Elkan, C. (2009) The Transporter Classification Database: recent advances. *Nucleic Acids Res*, **37**, D274-278.
8. Becnel, L.B., Darlington, Y.F., Ochsner, S.A., Easton-Marks, J.R., Watkins, C.M., McOwiti, A., Kankanamge, W.H., Wise, M.W., DeHart, M., Margolis, R.N. *et al.* (2015) Nuclear Receptor Signaling Atlas: Opening Access to the Biology of Nuclear Receptor Signaling Pathways. *PLoS One*, **10**, e0135615.
9. Wishart, D.S., Feunang, Y.D., Marcu, A., Guo, A.C., Liang, K., Vazquez-Fresno, R., Sajed, T., Johnson, D., Li, C., Karu, N. *et al.* (2018) HMDB 4.0: the human metabolome database for 2018. *Nucleic Acids Res*, **46**, D608-D617.
10. Wishart, D.S., Guo, A., Oler, E., Wang, F., Anjum, A., Peters, H., Dizon, R., Sayeeda, Z., Tian, S., Lee, B.L. *et al.* (2022) HMDB 5.0: the Human Metabolome Database for 2022. *Nucleic Acids Res*, **50**, D622-D631.
11. Wishart, D.S., Jewison, T., Guo, A.C., Wilson, M., Knox, C., Liu, Y., Djoumbou, Y., Mandal, R., Aziat, F., Dong, E. *et al.* (2013) HMDB 3.0--The Human Metabolome Database in 2013. *Nucleic Acids Res*, **41**, D801-807.
12. Wishart, D.S., Tzur, D., Knox, C., Eisner, R., Guo, A.C., Young, N., Cheng, D., Jewell, K., Arndt, D., Sawhney, S. *et al.* (2007) HMDB: the Human Metabolome Database. *Nucleic Acids Res*, **35**, D521-526.

13. Thiele, I., Swainston, N., Fleming, R.M., Hoppe, A., Sahoo, S., Aurich, M.K., Haraldsdottir, H., Mo, M.L., Rolfsson, O., Stobbe, M.D. *et al.* (2013) A community-driven global reconstruction of human metabolism. *Nat Biotechnol*, **31**, 419-425.
14. Husted, A.S., Trauelsen, M., Rudenko, O., Hjorth, S.A. and Schwartz, T.W. (2017) GPCR-Mediated Signaling of Metabolites. *Cell Metab*, **25**, 777-796.
15. Kooistra, A.J., Mordalski, S., Pandy-Szekeres, G., Esguerra, M., Mamyrbekov, A., Munk, C., Keseru, G.M. and Gloriam, D.E. (2021) GPCRdb in 2021: integrating GPCR sequence, structure and function. *Nucleic Acids Res*, **49**, D335-D343.
16. Safran, M., Dalah, I., Alexander, J., Rosen, N., Iny Stein, T., Shmoish, M., Nativ, N., Bahir, I., Doniger, T., Krug, H. *et al.* (2010) GeneCards Version 3: the human gene integrator. *Database (Oxford)*, **2010**, baq020.
17. Pandy-Szekeres, G., Esguerra, M., Hauser, A.S., Caroli, J., Munk, C., Pilger, S., Keseru, G.M., Kooistra, A.J. and Gloriam, D.E. (2022) The G protein database, GproteinDb. *Nucleic Acids Res*, **50**, D518-D525.
18. Moore, J.H. (1999) Bootstrapping, permutation testing and the method of surrogate data. *Phys Med Biol*, **44**, L11-12.
19. Hochberg, Y.B.a.Y. (1995) Controlling the False Discovery Rate: a Practical and Powerful Approach to Multiple Testing. *J. R. Statist. Soc*, **57**, 289-300.
20. Orth, J.D., Thiele, I. and Palsson, B.O. (2010) What is flux balance analysis? *Nat Biotechnol*, **28**, 245-248.
21. Wagner, A., Wang, C., Fessler, J., DeTomaso, D., Avila-Pacheco, J., Kaminski, J., Zaghouani, S., Christian, E., Thakore, P., Schellhaass, B. *et al.* (2021) Metabolic modeling of single Th17 cells reveals regulators of autoimmunity. *Cell*, **184**, 4168-4185 e4121.
22. Purohit, V., Wagner, A., Yosef, N. and Kuchroo, V.K. (2022) Systems-based approaches to study immunometabolism. *Cell Mol Immunol*, **19**, 409-420.
23. Alghamdi, N., Chang, W., Dang, P., Lu, X., Wan, C., Gampala, S., Huang, Z., Wang, J., Ma, Q., Zang, Y. *et al.* (2021) A graph neural network model to estimate cell-wise metabolic flux using single-cell RNA-seq data. *Genome Res*, **31**, 1867-1884.
24. Damiani, C., Maspero, D., Di Filippo, M., Colombo, R., Pescini, D., Graudenzi, A., Westerhoff, H.V., Alberghina, L., Vanoni, M. and Mauri, G. (2019) Integration of single-cell RNA-seq data into population models to characterize cancer metabolism. *PLoS Comput Biol*, **15**, e1006733.

25. Armingol, E., Ghaddar, A., Joshi, C.J., Baghdassarian, H., Shamie, I., Chan, J., Her, H.L., Berhanu, S., Dar, A., Rodriguez-Armstrong, F. *et al.* (2022) Inferring a spatial code of cell-cell interactions across a whole animal body. *PLoS Comput Biol*, **18**, e1010715.
26. Cang, Z., Zhao, Y., Almet, A.A., Stabell, A., Ramos, R., Plikus, M.V., Atwood, S.X. and Nie, Q. (2023) Screening cell-cell communication in spatial transcriptomics via collective optimal transport. *Nat Methods*.
27. Liu, Z., Sun, D. and Wang, C. (2022) Evaluation of cell-cell interaction methods by integrating single-cell RNA sequencing data with spatial information. *Genome Biol*, **23**, 218.
28. Asp, M., Giacomello, S., Larsson, L., Wu, C., Fürth, D., Qian, X., Wärdell, E., Custodio, J., Reimegård, J., Salmén, F. *et al.* (2019) A Spatiotemporal Organ-Wide Gene Expression and Cell Atlas of the Developing Human Heart. *Cell*, **179**, 1647-1660.e1619.
29. Fawkner-Corbett, D., Antanaviciute, A., Parikh, K., Jagielowicz, M., Geros, A.S., Gupta, T., Ashley, N., Khamis, D., Fowler, D., Morrissey, E. *et al.* (2021) Spatiotemporal analysis of human intestinal development at single-cell resolution. *Cell*, **184**, 810-826 e823.
30. Moncada, R., Barkley, D., Wagner, F., Chiodin, M., Devlin, J.C., Baron, M., Hajdu, C.H., Simeone, D.M. and Yanai, I. (2020) Integrating microarray-based spatial transcriptomics and single-cell RNA-seq reveals tissue architecture in pancreatic ductal adenocarcinomas. *Nat Biotechnol*, **38**, 333-342.
31. Ji, A.L., Rubin, A.J., Thrane, K., Jiang, S., Reynolds, D.L., Meyers, R.M., Guo, M.G., George, B.M., Mollbrink, A., Bergenstrahle, J. *et al.* (2020) Multimodal Analysis of Composition and Spatial Architecture in Human Squamous Cell Carcinoma. *Cell*, **182**, 497-514 e422.
32. Ru, B., Huang, J., Zhang, Y., Aldape, K. and Jiang, P. (2023) Estimation of cell lineages in tumors from spatial transcriptomics data. *Nat Commun*, **14**, 568.
33. Dunn, K.W., Kamocka, M.M. and McDonald, J.H. (2011) A practical guide to evaluating colocalization in biological microscopy. *Am J Physiol Cell Physiol*, **300**, C723-742.
34. Shamsi, F., Zheng, R., Ho, L.L., Chen, K. and Tseng, Y.H. (2023) Comprehensive analysis of intercellular communication in the thermogenic adipose niche. *Commun Biol*, **6**, 761.
35. Shamsi, F., Piper, M., Ho, L.L., Huang, T.L., Gupta, A., Streets, A., Lynes, M.D. and Tseng, Y.H. (2021) Vascular smooth muscle-derived Trpv1(+) progenitors are a source of cold-induced thermogenic adipocytes. *Nat Metab*, **3**, 485-495.

36. Wolf, F.A., Angerer, P. and Theis, F.J. (2018) SCANPY: large-scale single-cell gene expression data analysis. *Genome Biol*, **19**, 15.
37. Becht, E., McInnes, L., Healy, J., Dutertre, C.A., Kwok, I.W.H., Ng, L.G., Ginhoux, F. and Newell, E.W. (2018) Dimensionality reduction for visualizing single-cell data using UMAP. *Nat Biotechnol*.
38. Franzen, O., Gan, L.M. and Bjorkegren, J.L.M. (2019) PanglaoDB: a web server for exploration of mouse and human single-cell RNA sequencing data. *Database (Oxford)*, **2019**.
39. Sun, D., Liu, Z., Li, T., Wu, Q. and Wang, C. (2022) STRIDE: accurately decomposing and integrating spatial transcriptomics using single-cell RNA sequencing. *Nucleic Acids Res*, **50**, e42.
40. Li, W., Xu, H., Xiao, T., Cong, L., Love, M.I., Zhang, F., Irizarry, R.A., Liu, J.S., Brown, M. and Liu, X.S. (2014) MAGeCK enables robust identification of essential genes from genome-scale CRISPR/Cas9 knockout screens. *Genome Biol*, **15**, 554.
41. Lachmann, A., Torre, D., Keenan, A.B., Jagodnik, K.M., Lee, H.J., Wang, L., Silverstein, M.C. and Ma'ayan, A. (2018) Massive mining of publicly available RNA-seq data from human and mouse. *Nat Commun*, **9**, 1366.
42. Tseng, Y.H., Kriauciunas, K.M., Kokkotou, E. and Kahn, C.R. (2004) Differential roles of insulin receptor substrates in brown adipocyte differentiation. *Mol Cell Biol*, **24**, 1918-1929.
43. Ntranos, A., Park, H.J., Wentling, M., Tolstikov, V., Amatruda, M., Inbar, B., Kim-Schulze, S., Frazier, C., Button, J., Kiebish, M.A. *et al.* (2022) Bacterial neurotoxic metabolites in multiple sclerosis cerebrospinal fluid and plasma. *Brain*, **145**, 569-583.
44. Want, E.J., Masson, P., Michopoulos, F., Wilson, I.D., Theodoridis, G., Plumb, R.S., Shockcor, J., Loftus, N., Holmes, E. and Nicholson, J.K. (2013) Global metabolic profiling of animal and human tissues via UPLC-MS. *Nat Protoc*, **8**, 17-32.
